# Flow cytometry identifies changes in peripheral and intrathecal lymphocyte patterns in CNS autoimmune disorders and primary CNS malignancies

**DOI:** 10.1186/s12974-024-03269-3

**Published:** 2024-11-04

**Authors:** Saskia Räuber, Andreas Schulte-Mecklenbeck, Alice Willison, Ramona Hagler, Marius Jonas, Duygu Pul, Lars Masanneck, Christina B. Schroeter, Kristin S. Golombeck, Stefanie Lichtenberg, Christine Strippel, Marco Gallus, Andre Dik, Ruth Kerkhoff, Sumanta Barman, Katharina J. Weber, Stjepana Kovac, Melanie Korsen, Marc Pawlitzki, Norbert Goebels, Tobias Ruck, Catharina C. Gross, Werner Paulus, Guido Reifenberger, Michael Hanke, Oliver Grauer, Marion Rapp, Michael Sabel, Heinz Wiendl, Sven G. Meuth, Nico Melzer

**Affiliations:** 1 Department of Neurology, Medical Faculty and University Hospital Düsseldorf, Heinrich Heine University Düsseldorf, 40225 Düsseldorf, Germany; 2 Department of Neurology with Institute of Translational Neurology, University of Münster, Münster, Germany; 3 German Cancer Consortium (DKTK), German Cancer Research Center (DKFZ), Heidelberg, Germany; 4 Neurological Institute (Edinger Institute), University Hospital, Goethe University, Frankfurt/Main, Germany; 5 Frankfurt Cancer Institute (FCI), Frankfurt/Main, Germany; 6 Institute of Neuropathology, University of Münster, Münster, Germany; 7 Institute of Neuropathology, Medical Faculty and University Hospital Düsseldorf, Heinrich Heine University Düsseldorf, Düsseldorf, Germany; 8 Institute of Neuroscience and Medicine, Brain and Behaviour (INM-7), Research Center Jülich, Jülich, Germany; 9 Institute of Systems Neuroscience, Medical Faculty and University Hospital Düsseldorf, Heinrich Heine University Düsseldorf, Düsseldorf, Germany; 10 Department of Neurosurgery, Medical Faculty and University Hospital Düsseldorf, Heinrich Heine University Düsseldorf, Düsseldorf, Germany

**Keywords:** Glioblastoma, Primary diffuse large B cell lymphoma of the CNS, Autoimmune limbic encephalitis, Relapsing–remitting multiple sclerosis, Multidimensional flow cytometry

## Abstract

**Background:**

Immune dysregulation is a hallmark of autoimmune diseases of the central nervous system (CNS), characterized by an excessive immune response, and primary CNS tumors (pCNS-tumors) showing a highly immunosuppressive parenchymal microenvironment.

**Methods:**

Aiming to provide novel insights into the pathogenesis of CNS autoimmunity and cerebral tumor immunity, we analyzed the peripheral blood (PB) and cerebrospinal fluid (CSF) of 81 autoimmune limbic encephalitis (ALE), 148 relapsing–remitting multiple sclerosis (RRMS), 33 IDH-wildtype glioma, 9 primary diffuse large B cell lymphoma of the CNS (CNS-DLBCL), and 110 controls by flow cytometry (FC). Additionally, an in-depth immunophenotyping of the PB from an independent cohort of 20 RRMS and 18 IDH-wildtype glioblastoma patients compared to 19 controls was performed by FC combined with unsupervised computational approaches.

**Results:**

We identified alterations in peripheral and intrathecal adaptive immunity, mainly affecting the T cell (Tc) but also the B cell (Bc) compartment in ALE, RRMS, and pCNS-tumors compared to controls. ALE, RRMS, and pCNS-tumors featured higher expression of the T cell activation marker HLA-DR, which was even more pronounced in pCNS-tumors than in ALE or RRMS. Glioblastoma patients showed signs of T cell exhaustion that were not visible in RRMS patients. In-depth characterization of the PB revealed differences mainly in the T effector and memory compartment between RRMS and glioblastoma patients and similar alterations in the Bc compartment, including atypical Bc, CD19^+^CD20^−^ double negative Bc, and plasma cells. PB and CSF mFC together with CSF routine parameters could reliably differentiate ALE and RRMS from pCNS-tumors facilitating early diagnosis and treatment.

**Conclusions:**

ALE, RRMS, and pCNS-tumors show distinct but partially overlapping changes mainly in HLA-DR^+^ Tc, memory Tc, exhausted Tc, and Bc subsets providing insights into disease pathogenesis. Moreover, mFC shows diagnostic potential facilitating early diagnosis and treatment.

**Graphical Abstract:**

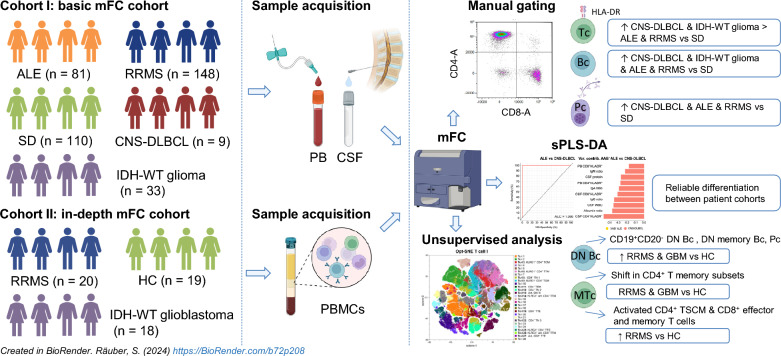

**Supplementary Information:**

The online version contains supplementary material available at 10.1186/s12974-024-03269-3.

## Introduction

A dysregulated immune response has been linked to different autoimmune diseases including those affecting the central nervous system (CNS).

Relapsing–remitting multiple sclerosis (RRMS) represents the prototypical chronic inflammatory CNS disease, primarily affecting the CNS white matter. In contrast, autoimmune limbic encephalitis (ALE) is an immune-mediated inflammatory condition predominantly affecting the gray matter of the CNS. Disease pathology is mainly confined to the limbic system, which accounts for the typical clinical presentation including temporal lobe seizures, memory deficits, and behavioral changes [1, 2]. A variety of autoantibodies in serum and/or cerebrospinal fluid (CSF) can be associated with ALE (referred to as antibody-positive ALE). In a considerable number of patients, the clinical manifestation is suggestive of ALE, nevertheless no autoantibodies can be detected in serum and CSF despite a thorough diagnostic workup (referred to as antibody-negative ALE). Diagnosis of antibody-negative ALE is especially challenging, delaying definite diagnosis and initiation of immunotherapy associated with worse outcomes [3–5].

The adaptive immune response seems to play a pivotal role in the pathogenesis of RRMS and ALE. Previous studies indicate that both (B and T cell) arms of the adaptive immune system are activated in ALE, however, the relative contribution to the local immunopathology in the brain parenchyma depends on the cellular localization of the target antigen [6–12]. Particularly, neural-antigen specific cytotoxic T cells (Tc)—as part of the adaptive immune system—can impair neuronal excitability and mediate neuronal dysfunction and degeneration. In this context, the release of cytokines, granzyme-B, and perforin, as well as ligation of death receptors are important mediators of cytotoxicity [5, 13]. Correlation of Tc with clinical parameters emphasizes their relevance during disease pathogenesis [11, 14]. Furthermore, the analysis of the Tc and B cell receptor repertoire revealed clonal expansion and restriction of B cells (Bc), plasma cells (Pc), and Tc mainly in the CSF of patients with ALE [15–17]. Differential expression analysis highlighted an upregulation of genes related to cytotoxicity and activation of Tc in expanded compared to non-expanded CSF Tc clones [17]. Expression of monoclonal antibodies from Bc receptor data confirmed autoantigen-reactivity [16, 17]. Likewise, single-cell RNA sequencing (sc-RNA-seq) identified clonally expanded CD8^+^ Tc, plasmablasts, and CD4^+^ Tc with an activated tissue-resident memory and cytotoxic phenotype in MS [18, 19]. Moreover, a disbalance between inflammatory T helper (Th) 17 and regulatory Tc (Treg) has been linked to disease pathology of MS [20].

In contrast to the excessive immune response observed in CNS autoimmune disorders, a highly immunosuppressive parenchymal microenvironment impairs an adequate anti-tumor T and B cellular immune response in primary CNS tumors (pCNS-tumors), such as isocitrate dehydrogenase (IDH)-wildtype glioblastoma and primary diffuse large B cell lymphoma of the CNS (CNS-DLBCL) [21]. In line, increased tumor infiltration of immune cells, especially Tc, has been associated with a prolonged survival of glioblastoma and lymphoma patients in some studies [21, 22]. Keeping the balance between immunosuppression and autoimmunity can be challenging as revealed by side effects of immune checkpoint inhibitors used for tumor treatment and immunomodulatory therapies for the treatment of autoimmune disorders [23–27].

From a clinical perspective, gliomas and CNS-DLBCL are possible differential diagnoses to ALE and also MS. Differentiating ALE from these tumors based on clinical features and results from technical studies can be challenging, especially if no autoantibodies are present (antibody-negative ALE) [28, 29]. In addition, gliomas and CNS-DLBCL may also mimic MS lesions on magnetic resonance imaging (MRI) [30–32]. Thus, invasive diagnostic tools are often necessary to solve the differential diagnosis.

Even though efforts have been made to characterize the immune cell pattern in patients with CNS tumors or CNS autoimmune diseases [11, 21, 22, 33–37], there are currently no studies providing an in-depth comparison of peripheral and intrathecal immune cell profiles between these diseases.

Thus, we performed a comprehensive study of the peripheral blood (PB) and CSF immune cell profiles of ALE and RRMS patients in comparison to patients with IDH-wildtype gliomas/glioblastomas, or CNS-DLBCL as well as to non-inflammatory controls aiming to (1) enhance the pathophysiological understanding of CNS autoimmunity and its relation to the anti-tumor immunity, and to (2) evaluate the diagnostic potential of multidimensional flow cytometry (mFC).

## Results

### Basic cohort characteristics

Two independent patient and control cohorts were recruited (cohort I: basic mFC cohort and cohort II: in-depth mFC cohort). In total, 309 patients and 129 controls were included in the study. Basic cohort characteristics are summarized in Table 1 and supplementary tables 2–4.

**Table 1:** Basic demographics and disease characteristics

	ALE	RRMS	IDH-WT glioma	CNS-DLBCL	SD	HC
Number of patients	81	168	51	9	110	19
Basic mFC analysis (PB & CSF)	81	148	33	9	110	0
In-depth mFC analysis (PBMCs)	0	20	18	0	0	19
Age (median with range) [years]	57 [17–80]	31 [15–61]	61 [20–86]	71 [61–90]	36 [15–79]	54 [45–69]
Sex [% female]	43.21	71.43	37.25	22.22	69.09	36.84
AABs [%]	Intra: 23.46 Extra: 24.69 Unknown: 2.47 AAB^−^: 49.38	NA	NA	NA	NA	NA
Immunotherapy within 4 weeks prior to sampling [%]	PLEX: 1.23 Steroids: 17.28	None	Steroids: 17.65	None	None	None

*AAB*^*−*^ autoantibody-negative, *AABs* autoantibodies, *ALE* autoimmune limbic encephalitis, *CSF* cerebrospinal fluid, *CNS-DLBCL* diffuse large B cell lymphoma of the central nervous system, *Extra* extracellular target epitope, *HC* healthy control, *IDH* isocitrate dehydrogenase, *Intra* intracellular target epitope, *mFC* multidimensional flow cytometry, *NA* not available, *PB* peripheral blood, *PBMCs* peripheral blood mononuclear cells, *PLEX* plasmapheresis, *RRMS* relapsing–remitting multiple sclerosis, *SD* somatic symptom disorder, *WT* wildtype

### Similarities in adaptive immunity between ALE and pCNS-tumors

**Fig. 1:**
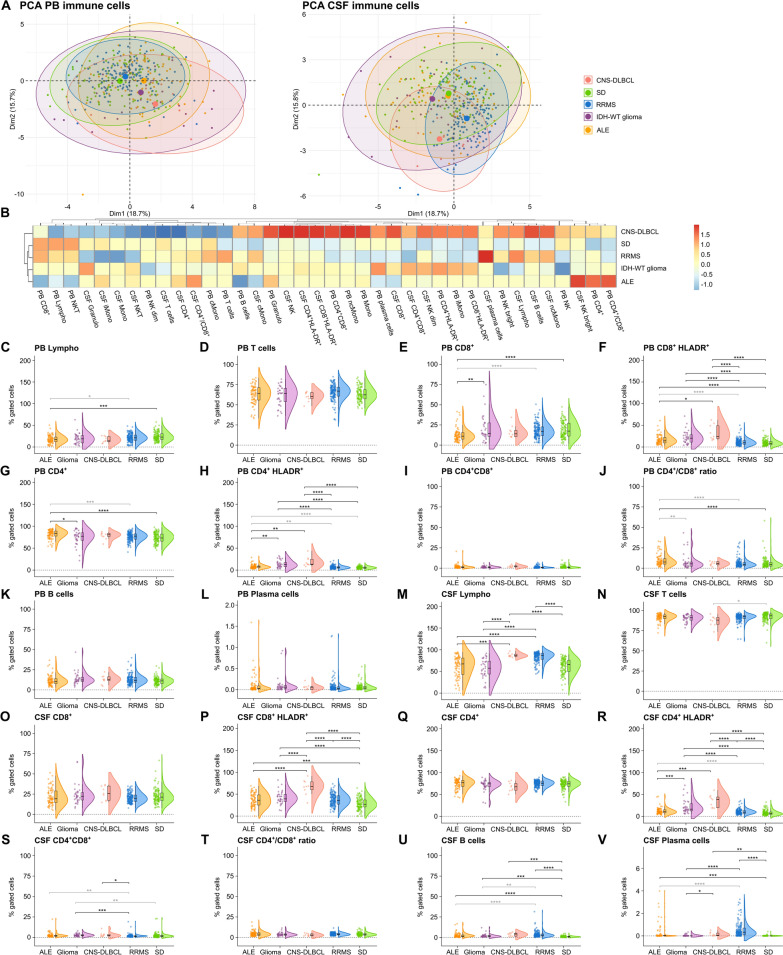
Pronounced adaptive immune response in ALE, IDH-wildtype glioma, and CNS-DLBCL compared to SD controls. **A** PCA including either PB mFC or CSF mFC parameters (% of gated cells) of ALE, RRMS, IDH-wildtype glioma, CNS-DLBCL, and SD patients. Every patient is displayed as a colored symbol.** B** Heatmap analysis of PB and CSF mFC parameters (% of gated cells) from ALE, RRMS, IDH-wildtype glioma, CNS-DLBCL, and SD patients: the median of each parameter was calculated, scaled, centered, and clustered hierarchically; **C**–**V** Violin plots with overlaying box plots depicting the PB and CSF mFC parameters of ALE, RRMS, IDH-wildtype glioma, CNS-DLBCL, and SD patients. Boxes display the median as well as the 25th and 75th percentiles. The whiskers extend from the hinge to the largest and smallest values, respectively, but no further than 1.5 * IQR from the hinge. P-values were calculated by ANOVA with post-hoc Tukey HSD if normality could be assumed based on Shapiro–Wilk test, otherwise Kruskal Wallis test with Dunn post hoc test (p-adjustment method: Benjamini–Hochberg) was used. *p ≤ 0.05, **p ≤ 0.01, ***p ≤ 0.001, ****p ≤ 0.0001. *ALE *autoimmune limbic encephalitis,* CD4*^+^*/CD8*^+^ CD4^+^/CD8^+^ ratio, *CNS *Central nervous system,* CSF *cerebrospinal fluid,* CNS-DLBCL *diffuse large B cell lymphoma of the central nervous system,* IDH *isocitrate dehydrogenase, *Lympho *lymphocytes,* mFC *multidimensional flow cytometry,* PB *peripheral blood,* PCA* principal component analysis, *RRMS *relapsing remitting multiple sclerosis,* SD *somatic symptom disorder*, WT *wildtype

Comparing the overall PB and CSF immune cell profiles between ALE, RRMS, IDH-WT glioma and CNS-DLBCL using principal component analysis (PCA), a marked overlap in PB immune cell profiles was noted between RRMS patients and somatic symptom disorder (SD) controls slightly different from ALE patients and distinct from IDH-WT glioma and CNS-DLBCL patients. Regarding the CSF, an overlap in the immune cell profile was seen between ALE patients and SD controls slightly different from IDH-WT glioma patients and distinct from CNS-DLBCL and RRMS patients (Fig. 1A).

To obtain a closer look at the different immune cell subsets, we created a heatmap including all PB and CSF mFC parameters as well as violin plots with overlaying box plots (Fig. 1B–V and supplementary Fig. 1).

In the PB, ALE patients had a shift from CD4^+^ to CD8^+^ Tc with an overall reduction of lymphocytes compared to SD controls (Fig. 1B, C, E, G, J). The fractions of activated CD4^+^ and CD8^+^ Tc were higher in the PB and CSF of ALE patients in relation to controls (Fig. 1B, F, H, P, R). Furthermore, a pronounced Bc and Pc response was observed in the CSF of ALE patients in relation to SD (Fig. 1B, U, V). CSF activated CD4^+^ Tc were confounded by differences in age and sex between groups (Fig. 1R).

Comparable to ALE, IDH-wildtype glioma patients were characterized by a pronounced PB and CSF adaptive immune response with an increase in activated CD4^+^ and CD8^+^ Tc in PB and CSF, and Bc in CSF (Fig. 1B, F, H, P, R, U).

Directly comparing ALE with IDH-WT glioma, activated CD4^+^ Tc were even higher in IDH-WT glioma than in ALE patients (Fig. 1B, H, R**)**, whereas CD4^+^ were elevated in the PB of ALE patients (Fig. 1B, G).

Comparing CNS-DLBCL and SD patients, we found increased fractions of activated CD4^+^ and CD8^+^ Tc in PB and CSF as well as CSF Bc and Pc, similar to ALE compared to SD (Fig. 1B, F, H, P, R, U, V). Furthermore, the overall percentage of CSF lymphocytes was higher in CNS-DLBCL compared to SD (Fig. 1B, M).

We next directly compared ALE with CNS-DLBCL and found higher percentages of CSF lymphocytes as well as activated CD4^+^ and CD8^+^ Tc in PB and CSF in CNS-DLBCL than in ALE patients (Fig. 1B, F, H, M, P, R).

**Fig. 2:**
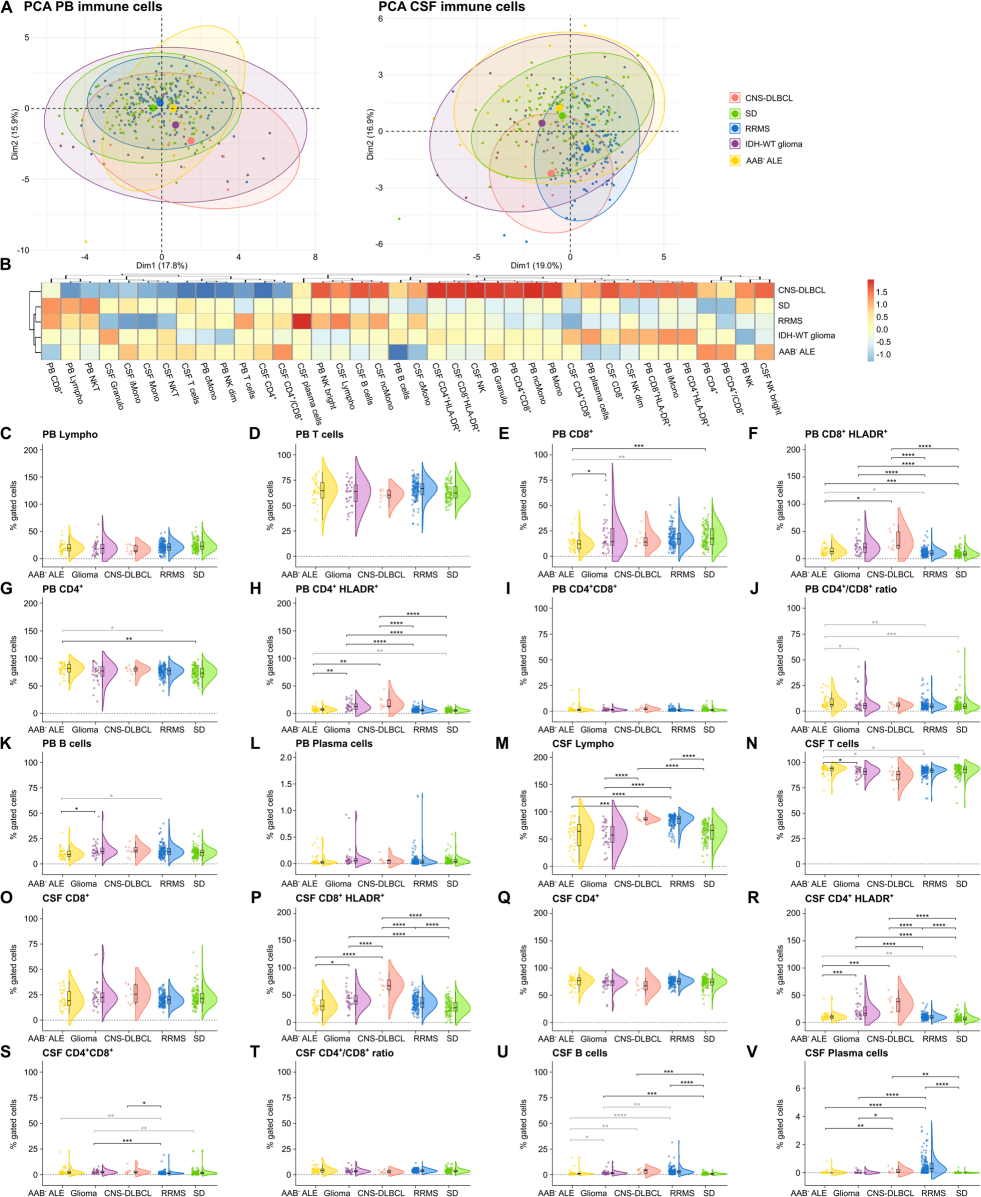
Antibody-negative ALE features similarities in adaptive immunity with IDH-wildtype glioma, and CNS-DLBCL. **A** PCA including either PB mFC or CSF mFC parameters (% of gated cells) of AAB^−^ ALE, RRMS, IDH-wildtype glioma, CNS-DLBCL, and SD patients. Every patient is displayed as a colored symbol. **B** Heatmap analysis of PB and CSF mFC parameters (% of gated cells) from AAB^−^ ALE, RRMS, IDH-wildtype glioma, CNS-DLBCL, and SD patients: the median of each parameter was calculated, scaled, centered, and clustered hierarchically; **C**–**V** Violin plots with overlaying box plots depicting the PB and CSF mFC parameters of AAB^−^ ALE, RRMS, IDH-wildtype glioma, CNS-DLBCL, and SD patients. The whiskers extend from the hinge to the largest and smallest values, respectively, but no further than 1.5 * IQR from the hinge. P-values were calculated by ANOVA with post-hoc Tukey HSD if normality could be assumed based on Shapiro–Wilk test, otherwise Kruskal Wallis test with Dunn post hoc test (p-adjustment method: Benjamini–Hochberg) was used. *p ≤ 0.05, **p ≤ 0.01, ***p ≤ 0.001, ****p ≤ 0.0001. AAB^−^ *ALE *Antibody-negative autoimmune limbic encephalitis*, CD4*^+^*/CD8*^+^ CD4^+^/CD8^+^ ratio,* CNS *Central nervous system,* CSF *cerebrospinal fluid,* CNS-DLBCL *diffuse large B cell lymphoma of the central nervous system,* IDH *isocitrate dehydrogenase, *Lympho lymphocytes; mFC *multidimensional flow cytometry, *PB *peripheral blood,* PCA *principal component analysis,* RRMS *relapsing remitting multiple sclerosis, *SD *somatic symptom disorder, *WT *wildtype

We next repeated the analyses only including antibody-negative (AAB^−^) ALE patients. PCA revealed slightly different PB immune cell profiles between antibody-negative ALE, RRMS patients and SD controls distinct from IDH-wildtype glioma and CNS-DLBCL patients (Fig. 2A). Overall CSF immune cell profiles differed between groups, antibody-negative ALE patients and SD controls showing a pronounced overlap in CSF immune cell profiles slightly different from IDH-WT glioma patients and distinct from CNS-DLBCL and RRMS patients (Fig. 2A).

When comparing antibody-negative ALE patients to SD controls, changes in PB adaptive immune cells were comparable to ALE patients in relation to SD (Fig. 2B, E, F, G, H, J). In the CSF, only activated CD4^+^ Tc were increased, however, this parameter was confounded by age and sex differences (Fig. 1B, R).

Comparing antibody-negative ALE and IDH-WT glioma patients, the percentage of CD8^+^ Tc in PB was lower and the fraction of Tc in CSF higher in antibody-negative ALE (Fig. 2B, E, N). In turn, activated CD4^+^ Tc in PB and CSF, activated CD8^+^ Tc in CSF, and Bc in PB and CSF were higher in IDH-WT glioma than in antibody-negative ALE (Fig. 2B, H, K, P, R, U). Differences in CSF Bc did not remain significant after correction for age and sex (Fig. 2U).

Antibody-negative ALE and CNS-DLBCL patients showed similar increases in activated CD4^+^ Tc in PB and CSF as well as CD8^+^ Tc in PB, although the percentages were even higher in CNS-DLBCL than in antibody-negative ALE (Fig. 2B, F, H, R). Elevated fractions of lymphocytes, activated CSF CD8^+^ Tc, Bc, and Pc were only noted in CNS-DLBCL compared to SD (Fig. 2B, M, P, U, V). In turn, antibody-negative ALE patients showed a shift from CD8^+^ Tc to CD4^+^ Tc in the PB, which was not visible in CNS-DLBCL patients (Fig. 1B, E, G, J).

Differences in the innate immune response were not as pronounced as in the adaptive immune response and are visualized in supplementary Fig. 1.

In summary, ALE, IDH-wildtype glioma, and CNS-DLBCL patients feature a pronounced adaptive immune response, mainly driven by activated Tc in CSF and/or PB, but also affecting the B and plasma cell compartment. Direct comparison of ALE with IDH-wildtype glioma and CNS-DLBCL patients revealed that Tc activation was even more prominent in patients with pCNS-tumors.

### RRMS and pCNS-tumors share intrathecal Bc and Tc responses

Comparing adaptive immune cell profiles between RRMS and SD patients, no significant differences could be noted in the PB while in the CSF lymphocytes, especially activated CD4^+^ and CD8^+^ Tc as well as Bc and Pc, were elevated in RRMS patients (Fig. 1B, M, P, R, U, V). Activated CD4^+^ and CD8^+^ Tc were increased in the PB of IDH-WT glioma but not in RRMS patients compared to SD controls (Fig. 1B, F, H). In the CSF, higher fractions of activated CD4^+^ and CD8^+^ Tc as well as Bc were shared between RRMS and IDH-WT glioma patients in comparison to SD controls (Fig. 1B, P, R, U). Activated CD4^+^ Tc were even higher in IDH-WT glioma whereas Bc were higher in RRMS patients (Fig. 1B, R, U). The latter were confounded by differences in age and sex. Increased CSF lymphocytes and Pc could only be detected in RRMS patients compared to SD controls (Fig. 1B, M, V**)**.

CNS-DLBCL patients had higher percentages of activated CD4^+^ and CD8^+^ Tc in the PB compared to SD and RRMS patients (Fig. 1B, F, H). In CSF, CNS-DLBCL and RRMS shared the increase in lymphocytes, especially activated CD4^+^ and CD8^+^ Tc, Bc, and Pc (Fig. 1B, M, P, R, U, V). Percentages of activated Tc were even higher in CNS-DLBCL compared to RRMS (Fig. 1B, P, R).

Furthermore, differences between RRMS and primary CNS malignancies could also be noted with regard to innate immunity which are illustrated in supplementary Fig. 1.

Taken together, RRMS and primary CNS malignancies share changes in the intrathecal Bc, Pc, and Tc responses while differences were noted with regard to innate and PB immune cell patterns. Similarities were more pronounced with CNS-DLBCL than with IDH-WT glioma.

### MFC shows diagnostic potential

Next, we assessed the discriminatory power of CSF routine as well as PB and CSF mFC parameters to differentiate ALE or RRMS from pCNS-tumor patients.

First, we analyzed the discriminatory ability of CSF routine parameters. Differentiating (antibody-negative) ALE and RRMS from IDH-WT glioma patients, moderate AUC values were achieved. However, CSF routine parameters could distinguish (antibody-negative) ALE and RRMS from CNS-DLBCL patients with relatively high AUC values (Supplementary Fig. 2 A-F).

**Fig. 3:**
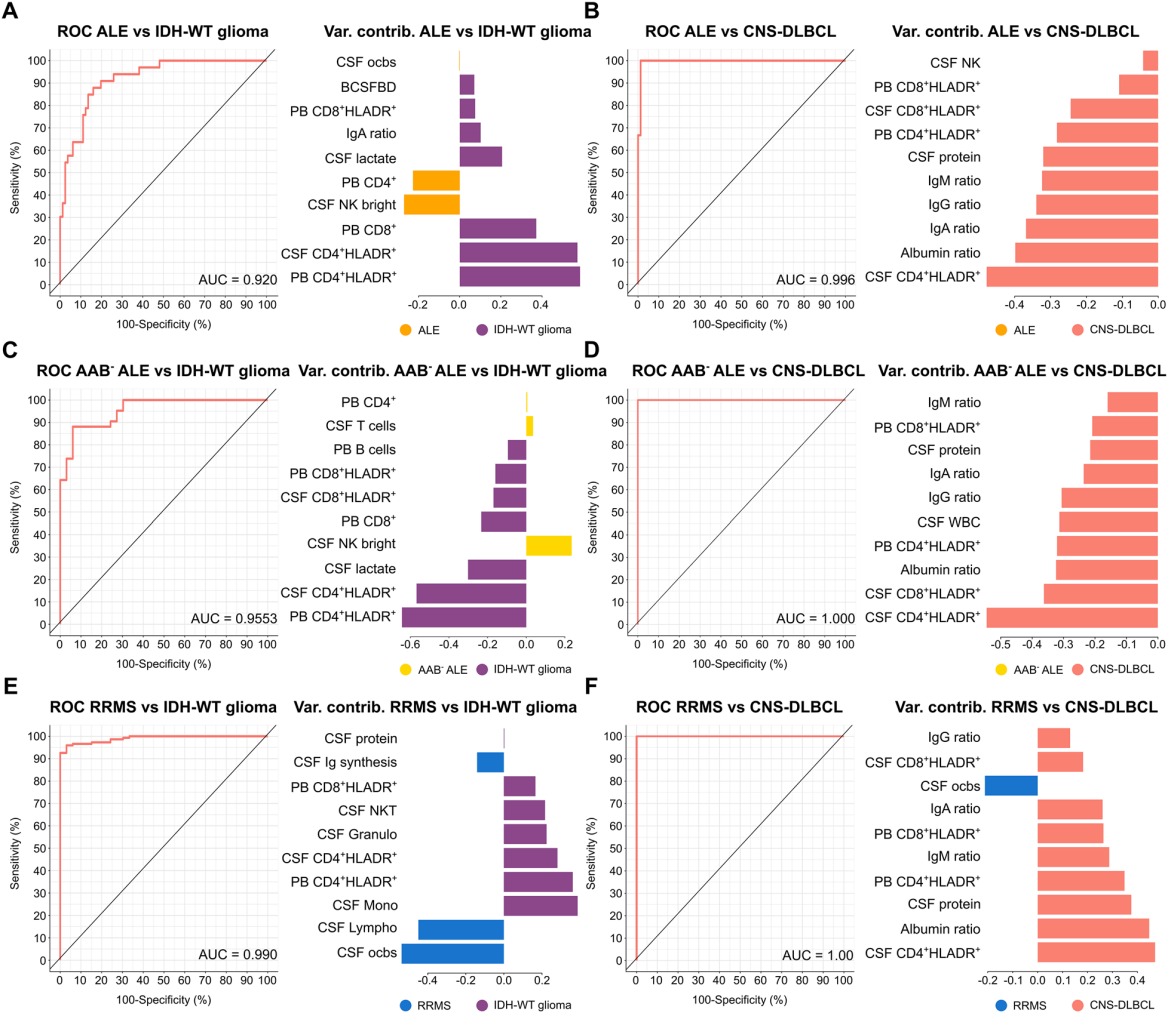
CSF routine together with PB and CSF mFC parameters can reliably differentiate ALE and RRMS patients from patients with primary CNS tumors. **A**–**F** ROC analyses of the classification results obtained from sPLS-DA including CSF routine, PB and CSF mFC parameters. ALE patients (**A** and **B**), antibody-negative ALE patients (**C** and **D**), or RRMS patients (**E** and **F**) were compared to either IDH-WT glioma or CNS-DLBCL. Loading plots visualize the top 10 variables contributing to latent component 1. Colors indicate the group in which the median is maximum. *AAB*^*−*^* ALE* antibody-negative ALE, *ALE* autoimmune limbic encephalitis, AUC Area under the curve, BCSFBD blood-CSF barrier dysfunction, cMono classical monocytes, *CSF* cerebrospinal fluid, *CNS-DLBCL* diffuse large B cell lymphoma of the central nervous system, *contrib* contribution, *IDH* isocitrate dehydrogenase wildtype glioma, *Granulo* granulocytes, *iMono* intermediate monocytes, *Lympho* lymphocytes, *mFC* multidimensional flow cytometry, *Mono* monocytes, *ncMono* non-classical monocytes, *NK* natural killer cells, *NKT* Natural killer T cells, *PB* peripheral blood, *ocbs* oligoclonal bands, *Q* ratio, *ROC* receiver operating characteristic, *RRMS* relapsing remitting multiple sclerosis, *SD* somatic symptom disorder, *sPLS-DA* Sparse Partial Least Squares Discriminant Analysis, *Var* variable *WBC* white blood cell count, *WT* wildtype

We next tested whether combining CSF routine with PB and CSF mFC analysis could increase the diagnostic accuracy. With this approach, ALE and IDH-WT glioma patients could be differentiated with an AUC of 0.920. PB/CSF CD4^+^HLADR^+^ and PB CD8^+^ were the most important parameters (Fig. 3A). When comparing ALE and CNS-DLBCL, a nearly perfect AUC (0.996) could be reached. CSF CD4^+^HLADR^+^, albumin ratio (QAlb), and immunoglobulin A ratio (QIgA) contributed the most to that model (Fig. 3B). Antibody-negative ALE could be distinguished from IDH-WT glioma patients with an AUC of 0.9553. PB/CSF CD4^+^HLADR^+^ and CSF lactate had the highest variable contribution (Fig. 3C). CNS-DLBCL could be perfectly differentiated from antibody-negative ALE patients (AUC of 1.0) with CSF CD4^+^HLADR^+^, CSF CD8^+^HLADR, and QAlb being the most important parameters (Fig. 3D). RRMS could be distinguished from IDH-WT glioma patients with an AUC of 0.990. CSF oligoclonal bands (ocbs), CSF lymphocytes, and CSF monocytes had the highest variable contribution (Fig. 3E). When differentiating RRMS and CNS-DLBCL patients, a perfect AUC (1.0) was achieved. CSF CD4^+^HLADR^+^, QAlb, and CSF protein contributed most to the model (Fig. 3F).

As lumbar puncture is an invasive procedure and cannot be performed in all patients, we also assessed the discriminatory ability of PB mFC parameters. This approach achieved AUC values between 0.836 and 0.969 but was inferior to the model including CSF routine and PB/CSF mFC parameters (Supplementary Fig. 2 G-L).

Overall, the combination of CSF routine with PB/CSF mFC analysis can reliably differentiate ALE and RRMS patients from patients with pCNS-tumors showing superiority over CSF routine analysis alone. PB mFC may be useful for patients who are ineligible for a lumbar puncture. Therefore, basic mFC analysis may be a beneficial complementary tool to the current diagnostic workup of CNS autoimmune diseases and pCNS-tumors to facilitate early diagnosis and guide treatment decisions.

### Reduction in several immune cell populations in the PB of glioblastoma patients

**Fig. 4:**
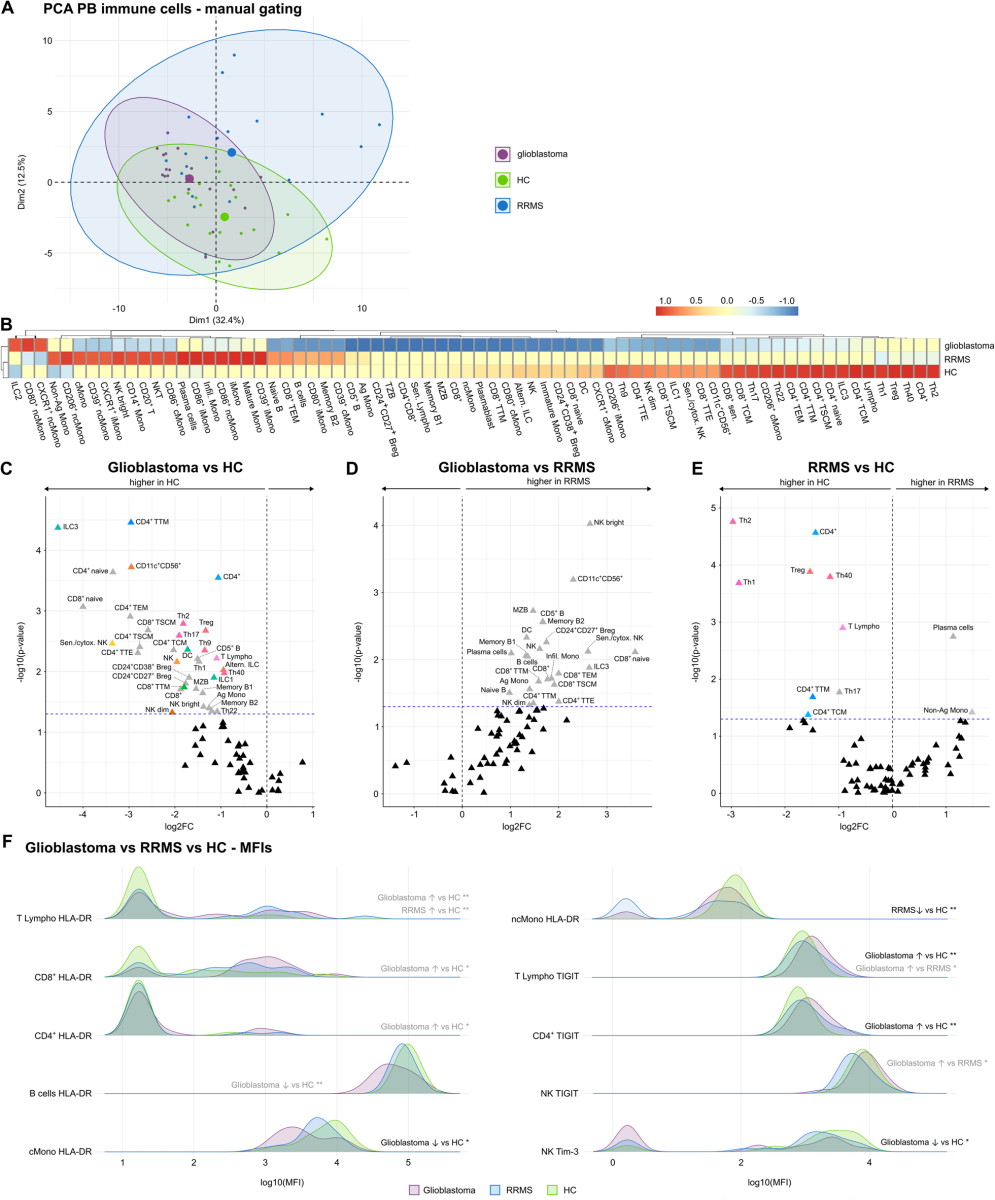
- Glioblastoma patients feature a reduction in innate and adaptive immune cell population in the PB and a higher abundance of cell activation and exhaustion markers. **A** PCA including PB mFC parameters (cell clusters as % of living cells identified by manual gating) of RRMS and glioblastoma patients as well as HC. Every patient is displayed as a colored symbol. **B** Heatmap analysis of PB mFC parameters (cell clusters as % of living cells identified by manual gating): the median of each parameter was calculated, scaled, centered, and clustered hierarchically. CD4^+^ senescent T cells are not visualized given the median of 0 in all groups; **C**–**E** Volcano plots showing PB mFC parameters of patients with glioblastoma or RRMS, and HC. The fold change of each single parameter between two groups is plotted against the corresponding p-value calculated by ANOVA with post-hoc Tukey HSD, if normality could be assumed based on Shapiro–Wilk test. Otherwise, Kruskal Wallis test with Dunn post hoc test (p-adjustment method: Benjamini–Hochberg) was used. Only significant parameters are labeled. Non-significant parameters are shown as black triangles. Parameters that did not remain significant after correction for age and sex are colored in grey. Senescent CD4^+^ are not visualized given the median of 0 in all groups. **F** Comparison of MFIs (medians) of different cell surface markers between patients with glioblastoma or RRMS and HC. *p ≤ 0.05, **p ≤ 0.01. *Ag* antigen-presenting, *Altern* alternative, *B* B cells, *Breg* B regulatory cells, *cMono* classical monocytes, *cytox* cytotoxic, *DC* dendritic cells, *HC* healthy control, *ILC* innate lymphoid cells, *iMono* intermediate monocytes, *Infil* infiltrating, *Lympho* lymphocytes, *mFC* multidimensional flow cytometry, *MFI* mean fluorescence intensity, *Mono* monocytes, *MZB* Marginal zone like B cells, *ncMono* non-classical monocytes, *NK* natural killer cells, *NKT* Natural killer T cells, *PCA* principal component analysis, *RRMS* relapsing remitting multiple sclerosis, *Sen* senescent, *T* T cells, *TCM *Central memory T cells, *TEM* Effector memory T cells, *Th* T helper cells, *Treg *Regulatory T cells, *TSCM* Stem memory T cells, *TTE* terminal effector T cells, *TZB* Transitional B cells

To further characterize the different immune cell subpopulations, we performed an in-depth analysis of glioblastoma compared to RRMS patients and healthy controls (HC). Given the limited availability of CSF from glioblastoma patients, PBMCs were used for the analysis. PCA revealed distinct peripheral immune cell profiles of glioblastoma, RRMS patients and controls with a slight overlap between glioblastoma and controls (Fig. 4A).

Detailed comparison of the PB immune cell profile between glioblastoma patients, HC, and RRMS patients identified a reduction in several innate and adaptive immune cell subsets in glioblastoma patients. Due to the age differences between patient cohorts, several parameters did not remain significant after correcting for age and sex (shown in grey) (Fig. 4B, C, D).

We next compared RRMS patients and HC. RRMS patients featured higher proportions of Pc and non-antigen-presenting monocytes. However, due to the age difference between the groups, those parameters were significantly confounded (Fig. 4B, E). Comparable to glioblastoma patients, the percentages of T lymphocytes and several T lymphocyte subsets were reduced in the PB of RRMS patients compared to HC (Fig. 4B, C, E).

Comparing the mean fluorescence intensities (MFI) of several markers on different immune cell populations between patient and control groups we could confirm our data from the basic mFC cohort showing that HLA-DR levels were more abundant on T lymphocytes of glioblastoma and RRMS patients compared to HC and on CD8^+^ and CD4^+^ lymphocytes of glioblastoma patients in comparison to HC. In contrast, HLA-DR abundance was reduced on Bc and cMono of glioblastoma patients in relation to HC and on non-classical monocytes (ncMono) of RRMS patients compared to HC (Fig. 4F). The cell exhaustion marker TIGIT was more abundant on lymphocytes of glioblastoma patients in comparison to HC and RRMS patients, on CD4^+^ lymphocytes of glioblastoma patients compared to HC, and on NK cells of glioblastoma in relation to RRMS patients. On the other hand, Tim-3 was less abundant on NK cells of glioblastoma patients compared to HC (Fig. 4F).

In summary, different innate and adaptive immune cell populations are reduced in the PB of glioblastoma compared to RRMS patients and HC. RRMS patients feature a reduction in several Th, T memory subsets, and Treg and an increase in the Bc and Pc response. Signs of cell activation were present in glioblastoma and RRMS patients and were even higher in glioblastoma patients while differences in cell exhaustion markers relative to HC were only visible in glioblastoma.

### Similarities in the Bc and differences in the Tc compartment between glioblastoma and RRMS patients

**Fig. 5:**
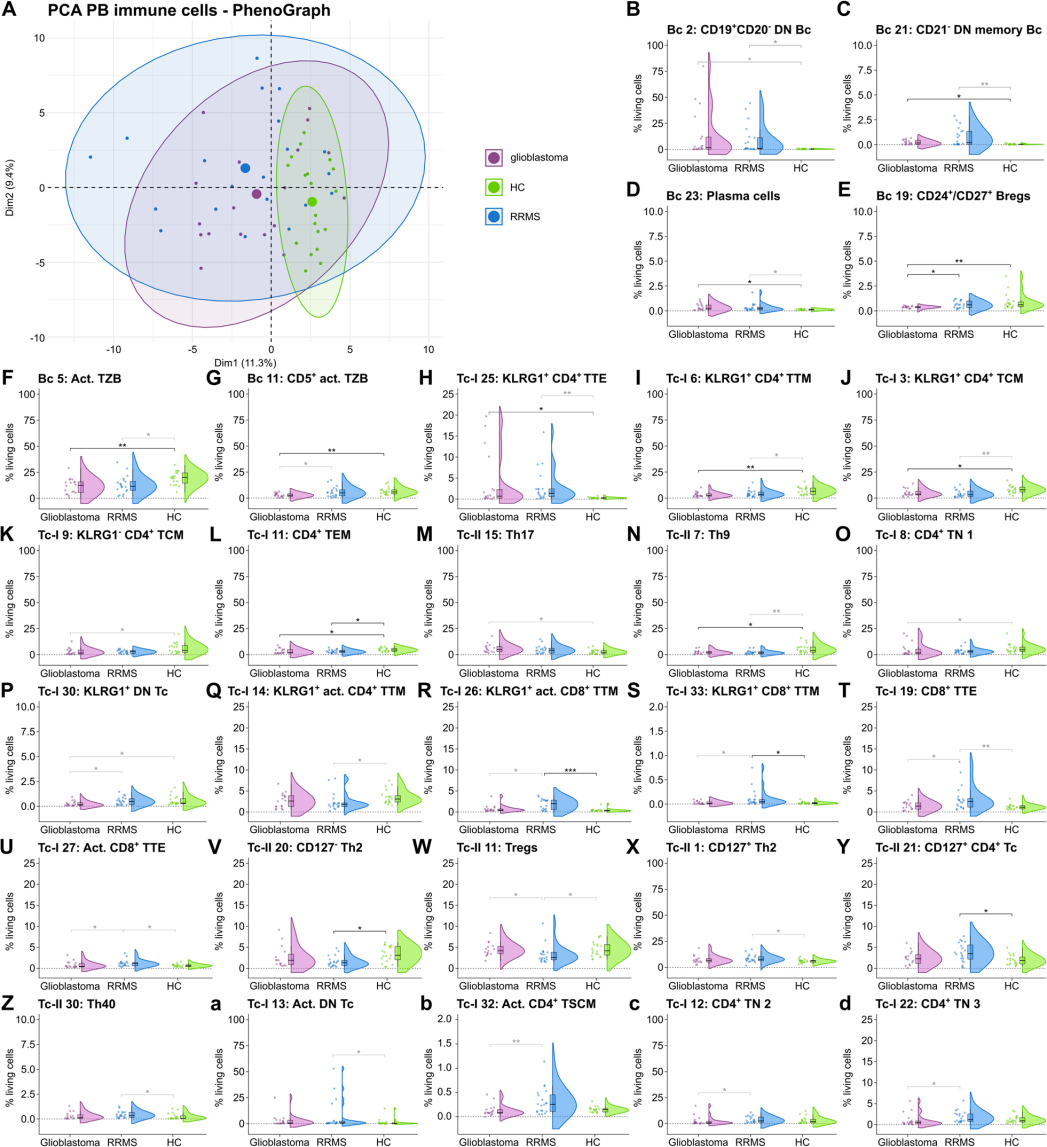
Unsupervised clustering identifies differences in T effector and memory subsets and similarities in the B cell compartment between glioblastoma and RRMS patients.** A** PCA including PB mFC parameters (cell clusters as % of living cells identified by PhenoGraph) of RRMS, glioblastoma patients, and HC. Every patient is displayed as a colored symbol. **B**–**d** Violin plots with overlaying box plots depicting the PB mFC parameters (cell clusters as % of living cells identified by PhenoGraph) of RRMS, glioblastoma patients, and HC. The whiskers extend from the hinge to the largest and smallest values, respectively, but no further than 1.5 * IQR from the hinge. P-values were calculated by ANOVA with post-hoc Tukey HSD if normality could be assumed based on Shapiro–Wilk test, otherwise Kruskal Wallis test with Dunn post hoc test (p-adjustment method: Benjamini–Hochberg) was used. *p ≤ 0.05, **p ≤ 0.01, ***p ≤ 0.001, ****p ≤ 0.0001. *Act. Activated, Bc—B cells, Bregs B regulatory cells; cMono—classical monocytes; DN Bc—double negative (CD27*^*−*^*IgD*^*−*^*) B cells, DN Tc double negative (CD4*^+^*CD8*^+^*) T cells, FC fold change, HC healthy control, mFC multidimensional flow cytometry, Mono monocytes, ncMono non-classical monocytes, PCA principal component analysis, RRMS relapsing remitting multiple sclerosis, Tc *T cells,* TCM *central memory T cells*, TEM *effector memory T cells,* Th *T helper cells,* TN *naïve T cells,* Tregs *regulatory T cells, *TSCM *stem memory T cells,* TTE *T terminal effector T cells,* TTM *transitional memory T cells,* TZB *transitional B cells

Finally, we performed an unsupervised analysis of the Bc, Tc, and monocyte populations (Supplementary Figs. 3–6). PCA revealed differences in the PB immune cell profile between glioblastoma, RRMS patients, and HC with an overlap between glioblastoma and RRMS patients (Fig. 5A).

Taking a closer look at the Bc clusters, higher percentages of CD19^+^CD20^−^ double negative (CD27^−^IgD^−^; DN) Bc (Bc 2), CD21^−^ DN memory Bc (Bc 21), and Pc (Bc 23) were found in glioblastoma patients compared to controls, whereas the fraction of CD24^+^/CD27^+^ regulatory Bc (Bregs) (Bc19) and (CD5^+^) activated transitional Bc (TZB) (Bc 5 & Bc 11) were reduced (Fig. 5B-G). Differences in Bc 2 did not remain significant after correction for age and sex (Fig. 5B). Likewise, RRMS patients featured higher percentages of CD19^+^CD20^−^ DN Bc (Bc 2), CD21^−^ DN memory Bc (Bc 21), and Pc (Bc 23) and a reduction in activated TZB (Bc 5). For the RRMS cohort, significance was not maintained after correction for age and sex imbalances. (Fig. 5B-D, F). Directly comparing glioblastoma and RRMS patients, higher fractions of CD5^+^ activated TZB (Bc 11) and CD24^+^/CD27^+^ Bregs (Bc 19) were noted in the PB of RRMS patients. The former were significantly confounded by age and sex differences (Fig. 5E, G).

Focusing on the Tc compartment, glioblastoma patients had a shift in the memory compartment with an increase in KLRG1^+^CD4^+^ terminal effector Tc (TTE; Tc-I 25) and a reduction in KLRG1^+^CD4^+^ transitional memory Tc (TTM; Tc-I 6), KLRG1^+^CD4^+^ central memory Tc (TCM; Tc-I 3), KLRG1^−^CD4^+^ TCM (Tc-I 9), and CD4^+^ effector memory Tc (TEM; Tc-I 11) compared to controls (Fig. 5H–L). Furthermore, Th17 (Tc-II 15) were higher and Th9 (Tc-II 7), CD4^+^ naïve Tc (Tc-I 8), and KLRG1^+^CD4^−^CD8^−^ Tc (DN Tc) (Tc-I 30) were lower in glioblastoma in comparison to controls. Nevertheless, some parameters were confounded by age and sex (Fig. 5M–P).

Comparable to glioblastoma patients, RRMS patients had higher fractions of KLRG1^+^CD4^+^ TTE (Tc-I 25) and reduced percentages of KLRG1^+^CD4^+^ TTM (Tc-I 6), KLRG1^+^CD4^+^ TCM (Tc-I 3), CD4^+^ TEM (Tc-I 11), and Th9 (Tc-II 7) compared to controls (Fig. 5H-J, L, N). Moreover, KLRG1^+^ activated CD4^+^ TTM (Tc-I 14) were reduced while KLRG1^+^ (activated) CD8^+^ TTM (Tc-I 26 & Tc-I 33), and (activated) CD8^+^ TTE (Tc-I 19 & Tc-I 27) were elevated in the PB of RRMS compared to controls (Fig. 5Q–U). In addition, a shift from CD127^−^ Th2 (Tc-II-20) and Tregs (Tc-II 11) towards CD127^+^ Th2 (Tc-II 1) and CD127^+^ CD4^+^ Tc (Tc-II 21), Th40 (Tc-II 30), and activated CD4^−^CD8^−^ Tc (Tc-I 13) was noted (Fig. 5V-a). However, several parameters were confounded by age and sex (Fig. 5H-a).

Comparing glioblastoma and RRMS patients, we detected a decrease in KLRG1^+^CD4^−^CD8^−^ Tc (Tc-I 30) in glioblastoma patients, also visible when comparing glioblastoma patients and controls (Fig. 5P). Tregs (Tc-II 11) were lower in RRMS and KLRG1^+^ (activated) CD8^+^ TTM (Tc-I-26 & Tc-I 33) and (activated) CD8^+^ TTE (Tc-I 19 & Tc-I 27) higher in RRMS in relation to glioblastoma patients similar to the comparison between RRMS patients and controls (Fig. 5R–U, W). What is more, activated CD4^+^ TSCM (Tc-I 32) and naïve CD4^+^ Tc (Tc-I 12 & Tc-I 22) were elevated in the PB of RRMS compared to glioblastoma patients (Fig. 5b–d). Due to imbalances in age and sex between groups, several parameters were confounded by age and sex (Fig. 5P, R–U, W, b–d).

Taken together, unsupervised clustering revealed similarities in the Bc compartment between RRMS and glioblastoma patients with an increase in CD19^+^CD20^−^ DN Bc, CD21^−^ DN memory Bc, and Pc as well as a reduction in activated TZB while CD24^+^/CD27^+^ Bregs were only reduced in glioblastoma patients compared to controls. In addition, glioblastoma and RRMS patients shared higher fractions of KLRG1^+^ CD4^+^ TTE, whereas CD8^+^ effector and memory Tc were only elevated in RRMS patients compared to controls. Furthermore, RRMS patients had higher fractions of activated CD4^+^ TSCM compared to glioblastoma patients.

## Discussion

Strict regulation of the immune system is crucial to obtain immune homeostasis [38]. A dysregulated immune response contributes to the pathogenesis of autoimmune inflammatory conditions of the CNS but is also involved in the immune response towards CNS tumors [1, 2, 21, 39]. In classical autoimmune inflammatory diseases of the CNS, ALE and RRMS among others, the immune system seems to be over-reactive targeting self-antigens leading to disruption of physiological processes [1, 2, 39, 40]. On the contrary, in pCNS-tumors a highly immunosuppressive microenvironment inhibits physiological immune mechanisms which confer tumor immunity, leading to failed control of tumor infiltration and progression [21]. The development of autoimmune disorders following immune checkpoint inhibition emphasizes the involvement of similar pathways in the pathogenesis of CNS autoimmune and neoplastic diseases [23, 24, 41–43]. This is further supported by the fact that genetic CTLA-4 deficiency triggers systemic autoimmunity [44]. Thus, a concise understanding of the pathways involved in the pathogenesis of CNS autoimmune diseases and pCNS-tumors as well as their regulation is crucial for the development of novel therapeutic approaches for both disease groups. Yet, studies relating the anti-tumor immune response to that underlying classical inflammatory CNS diseases remain scarce.

Analyzing the peripheral and intrathecal immune cell profile applying mFC, we were able to identify changes mainly in the adaptive immune response in patients with ALE, RRMS, or pCNS-tumors when compared to controls.

In the PB, ALE, IDH-WT glioma, and CNS-DLBCL patients shared an increase in activated CD8^+^ and CD4^+^ Tc, however, Tc activation was even more pronounced in IDH-WT glioma and CNS-DLBCL than in ALE. ALE patients had a shift from CD8^+^ Tc to CD4^+^ Tc, which was not visible in the other groups. Furthermore, in-depth phenotyping of PB immune cells also revealed changes in the effector and memory Tc compartment with similarities in KLRG1^+^ CD4^+^ TTE between RRMS and glioblastoma patients and differences in CD8^+^ effector and memory Tc and activated CD4^+^ TSCM. Regarding the Bc and Pc compartment, RRMS and glioblastoma patients shared an increase in CD19^+^CD20^−^ DN Bc, CD21^−^ DN memory Bc, and Pc as well as a reduction in activated TZB while CD24^+^/CD27^+^ Bregs were only reduced in glioblastoma patients compared to controls.

In the CSF, higher fractions of activated CD8^+^ and CD4^+^ Tc were observed in ALE, RRMS, IDH-WT glioma, and CNS-DLBCL patients. Again, T cell activation was more prominent in pCNS-tumors than in ALE or RRMS patients. Moreover, higher percentages of Bc were shared between ALE, RRMS, and pCNS-tumors, whereas Pc were only increased in ALE, RRMS, and CNS-DLBCL patients compared to controls.

Tc—as an important part of the adaptive immune system—are crucial in mediating host defense to pathogens and neoplastic cells [21]. However, an excessive Tc response can lead to neuronal damage negatively affecting neuronal integrity and excitability [5, 13]. Previous studies emphasized the pathophysiological role of Tc in ALE as well as in RRMS and the importance of Tc mediated anti-tumor immunity [11–13, 18–21, 33, 45–48]. In line with those observations, our basic mFC analysis identified changes in peripheral and intrathecal adaptive immunity, mainly affecting activated Tc. In CNS autoimmune disorders, activated lymphocytes exert a direct or indirect cytotoxic anti-neuronal immune response causing neuronal damage and impairing neuronal function [13]. In this context, we previously found elevated fractions of HLA-DR^+^ CD4^+^ and CD8^+^ Tc in PB and CSF of a small cohort of patients with GAD65-ALE. CSF CD8^+^ Tc negatively correlated with hippocampal volume and memory function emphasizing their pathophysiological relevance [35]. With regard to glioma patients, previous studies detected higher levels of HLA-DR in tumor tissue compared to normal tissue and elevated HLA-DR expression was associated with a lower survival rate [49]. Another study reported an increase in activated HLA-DR^+^CD8^+^ Tc in the PB and in tumor tissue of glioma patients, which was associated with disease progression, and higher percentages of this cell population were detected in high-grade as compared to low-grade gliomas. Furthermore, increased levels of the Tc exhaustion marker PD-1 were reported on HLA-DR^+^CD8^+^ Tc of glioma patients [50]. Even though we could not detect higher levels of PD-1 on lymphocytes in glioblastoma patients, these cells displayed higher TIGIT-MFIs, which presents another common Tc exhaustion marker, compared to controls. Collectively, these data imply that Tc are activated in glioma patients but display a functionally exhausted state hampering anti-tumor immunity. Similar increases in HLA-DR expression levels on CD4^+^ and CD8^+^ Tc in PB and CSF were visible in the CNS-DLBCL cohort. Data on HLA-DR expression on immune cells from CNS-DLBCL patients are scarce, however, single-cell analysis of tumor tissue-derived cells revealed an increase of Tc with a proliferating, activated, and exhaustive phenotype [51]. Taken together, HLA-DR^+^ Tc, e.g., via the modulation of Tc exhaustion, may be a promising therapeutic target in CNS autoimmune diseases and pCNS-tumors that warrants further investigation.

In addition to changes in activated Tc, in-depth phenotyping of the PB revealed reduction of several immune cell populations in glioblastoma compared to HC. This observation might imply systemic immune cell dysfunction in glioblastoma as previously reported [52, 53]. Chongsathidkiet et al. has described a sequestration of Tc in the bone marrow of patients with glioblastoma, which was associated with a tumor-induced reduction of S1P1 on the Tc surface [52]. In line with that, Ayasoufi et al. found signs of systemic immunosuppression affecting the cellular but also the humoral compartment and suggests circulating factors as treatment target to restore immunity [53]. However, as no data regarding the intrathecal and intraparenchymal immune response were available for our in-depth glioblastoma cohort, we cannot rule out recruitment of certain immune cell subsets to the CNS, which is (taking into consideration previous data [54–57]) likely to account for the reduction in several CD4^+^ Tc subsets in the PB of our in-depth RRMS cohort. Thus, future studies should include different immune cell compartments to provide clarification.

Beyond that, unsupervised clustering identified changes predominantly in the T effector and memory compartment in glioblastoma and RRMS patients. Memory Tc mediate long-term protection against pathogens but also solid tumors. Upon antigen re-encounter, they proliferate rapidly and execute cytotoxic functions [58]. Memory Tc have been reported to be implicated in several autoimmune disorders, MS and type 1 diabetes, among others. In this context, an enrichment of activated effector memory CD8^+^ Tc and an oligoclonal expansion of memory CD8^+^ Tc were detected in the CSF of MS patients [59, 60]. Within the brain parenchyma, invasion of tissue-resident memory Tc was observed [61]. Myelin-reactive Tc from MS patients were found to be more likely in the memory population, while myelin-reactive Tc from HC mainly showed a naïve phenotype [62]. In type-1 diabetes, an autoimmune stem-like CD8^+^ Tc population was found to be significantly involved in disease pathogenesis [63, 64]. In accordance with these findings, we detected higher fractions of CD8^+^ memory and effector Tc (KLRG1^+^ (activated) CD8^+^ TTM, and (activated) CD8^+^ TTE) in RRMS patients compared to HC. Effector and memory Tc within the CD4^+^ Tc compartment were mainly reduced in relation to controls, except for KLRG1^+^ CD4^+^ TTE. Furthermore, direct comparison between RRMS and glioblastoma patients identified an increase in activated CD4^+^ TSCM in RRMS patients. It is intriguing to speculate that those TSCM drive CNS autoimmunity by continuously providing help to CD8^+^ Tc. In contrast, glioblastoma patients were mainly characterized by lower fractions of several T memory subsets, except for KLRG1^+^ CD4^+^ TTE, which were more abundant in the PB of glioblastoma patients in comparison to controls. In general, dysregulation of the T effector and memory compartment seems to be crucially involved in the pathogenesis of CNS autoimmune disorders and pCNS-tumors. Limiting the autoreactive function of T effector and memory cells in CNS autoimmune disorders and revitalizing the effector functions of those cells in pCNS-tumors might thus yield therapeutic potential in the future.

Apart from a pronounced Tc response, our data hint towards an activation of the Bc arm of the adaptive immune system in the CSF and/or PB of ALE, RRMS, and pCNS-tumors. Bc are involved in antibody production by differentiation into plasmablasts and Pc [65]. In addition, they directly interact with Tc leading to Tc activation and clonal expansion by presenting antigens on their cell surface via MHCI and MHCII and providing costimulatory signals [66]. In ALE patients, especially those with autoantibodies against cell surface neural antigens, B and plasma cell infiltrates can be detected in the CNS and autoantibodies produced in the Bc compartment are deemed to exert direct pathogenic effects impairing synaptic transmission and leading to neural dysfunction [5, 9, 10, 67]. In pCNS-tumors, Bc can act as antigen presenting cells shaping the anti-tumor Tc response and produced antibodies can drive antibody-dependent cytotoxicity, phagocytosis, and complement activation [66, 68]. Depending on the microenvironment and phenotype, Bc can also mediate protumor effects [68]. For example, Breg seem to exert immunosuppressive activity towards activated CD8^+^ Tc by overexpressing inhibitory molecules, such as PD-L1 and CD155, and release of immunosuppressive cytokines (e.g., TGFβ and IL10) [69]. In-depth immunophenotyping in our cohort of glioblastoma patients showed decreased fractions of several Bc subsets including Bregs. Reduction of Bc subsets could be a sign of a dampened immune response in glioblastoma patients or be due to recruitment to the tumor microenvironment, as discussed above. Thus, it would be interesting to assess the detailed intrathecal and intraparenchymal Bc response in glioblastoma compared to RRMS patients and controls to improve our understanding of the role of Bc in CNS autoimmunity and in anti-tumor immunity. Interestingly, unsupervised cluster analysis revealed an increase in CD21^−^ DN memory Bc in the PB of glioblastoma and RRMS patients compared to controls. These Bc, which are also termed atypical Bc (aBc), have been previously reported to be expanded in autoimmune diseases, including RRMS [70]. ABc were found to be hyperresponsive to toll-like receptor signaling and differentiate without BCR stimulation into auto-reactive antibody-producing Pc [71, 72]. In tumor patients, aBc were elevated in the PB of patients with breast cancer [73] and correlated with lack of response to checkpoint inhibitors in patients with non-small-cell lung cancer [74]. Beyond that, an exhausted phenotype of those aBc, as a results of chronic antigen exposure, was reported in chronic infections [75–78]. In tumor patients, aBc are chronically exposed to tumor-antigens, so it is conceivable that they have functional similarities to aBc seen in chronic infection [74]. Furthermore, aBc are capable of antigen presentation to CD4^+^ Tc inducing a regulatory phenotype [79, 80]. In this regard, a lack of CD21 expression on Bc seems to hamper an effective anti-tumor immune response [74, 80]. Additional studies are necessary to provide further clarification regarding the exact phenotype and function of aBc in pCNS-tumors and CNS autoimmune diseases and to assess their therapeutic potential. Regarding the plasma cell compartment, there was an increase in Pc in the PB of glioblastoma and RRMS patients in comparison to controls. In MS, Pc were previously found to be expanded in the CSF and have been linked to disease pathology [19, 81]. Likewise, a plasma cell expansion was detected in the spleen and blood of mice with glioblastoma [82]. Of note, CD19^+^CD20^−^ DN Bc were also elevated in the PB of glioblastoma and RRMS patients compared to controls. Previous studies reported that a loss a of CD20 expression on human Bc was accompanied by a transient Bc activation inducing a Bc to Pc differentiation [83]. This mechanism might contribute to the increase in Pc seen in glioblastoma and RRMS patients. However, further studies are needed to elucidate this phenomenon and to assess the exact phenotype and functional state, especially antibody production, of those Pc in glioblastoma and RRMS. Based on those findings, Pc engineering might be an interesting field of study to identify potential novel therapeutic approaches [84].

Apart from providing mechanistic insights into to pathophysiology of diseases, mFC might also provide diagnostic benefits. Clinical diagnosis of ALE can be difficult in certain cases, especially if no autoantibodies can be detected in serum and CSF. Gliomas and CNS-DLBCL can mimic ALE based on clinical and radiological features, thus being important differential diagnoses to ALE [28, 29]. Concerning RRMS, differentiating inflammatory lesions from pCNS-tumors can also present a challenge under certain circumstances, especially tumefactive MS lesions can mimic pCNS-tumors [30–32, 85]. Based on our results, the combination of CSF routine with PB/CSF mFC analysis can reliably differentiate ALE and RRMS patients from patients with pCNS-tumors showing superiority over CSF routine analysis alone. Even in the PB, which can be obtained easily and presents a minimally invasive procedure, mFC parameters show a high discriminatory power when differentiating ALE and RRMS from pCNS-tumors, especially from CNS-DLBCL. Thus, mFC could facilitate the differential diagnosis of ALE, RRMS, and pCNS-tumors and could support the identification of patients benefiting from an immediate invasive workup.

Our study is limited by the imbalanced age- and sex ratios, the small patient numbers in some groups, and the pre-treatment of some glioblastoma patients with oral steroids. As a lumbar puncture is an invasive procedure and is often not part of the routine workup of pCNS-tumors, sample collection was prolonged and CSF samples were not available for all glioblastoma patients. As the diagnostic criteria of CNS tumors were updated in 2021, not all patients were diagnosed according to the current diagnostic criteria. Furthermore, no comparison of the intraparenchymal immune response could be performed as fresh brain samples of patients with CNS autoimmune disorders are very scarce. We are fully aware that IDH-wildtype glioma and CNS-DLBCL present heterogenous disease entities with different histopathology and molecular markers. Moreover, ALE patients with different target antigens as well as antibody-negative patients have been included. As a result of the small sample size, no further division into subgroups, depicting those heterogeneities, was performed. In addition, disease duration, genetic background, comorbidities, and lifestyle might also impact peripheral and intrathecal immune cell profiles, which might affect reproducibility of our results. Regarding the RRMS cohorts, differences in the immune cell profile between relapse and remission can also impact our results. Thus, additional studies with larger and deeply phenotyped groups of patients will be needed to validate our data.

In summary, ALE, RRMS, and pCNS-tumors are mainly characterized by changes in the adaptive immune response including signs of Tc activation in ALE, RRMS, and pCNS-tumors, Tc exhaustion in pCNS-tumors, in T effector and memory subsets and also in the Bc compartment. MFC was able to reliably differentiate ALE and RRMS from pCNS-tumors, which may facilitate clinical diagnosis and provide novel approaches for treatment to improve outcomes.

## Methods

### Study participant details

271 patients (81 ALE, 148 RRMS, 33 IDH-wildtype glioma, 9 CNS-DLBCL) who received PB and CSF mFC since 2012 were retrospectively identified from the local database of the Department of Neurology with Institute of Translational Neurology at the University Hospital Münster of the University of Münster, Germany, and were included in the basic mFC cohort. 110 patients with SD served as non-inflammatory and non-neoplastic controls (referred to as controls throughout the manuscript).

To perform an in-depth analysis of different immune cell subpopulations, 18 IDH-wildtype glioblastoma patients, 20 RRMS patients, and 19 healthy HC with available peripheral blood mononuclear cells (PBMCs) were identified from the local database of the Department of Neurology at the University Hospital of the Heinrich Heine University Düsseldorf, Germany, and were included in the in-depth mFC cohort.

All ALE patients were diagnosed according to the current, recently validated, diagnostic consensus criteria [39, 86] (Supplementary Table 1). None of the ALE patients received any immunotherapy at the time of sampling. Patients who were treated with any long-term immunotherapy (e.g., rituximab, azathioprine, and cyclophosphamide) prior to sampling were excluded. Basic demographic and clinical characteristics of ALE patients are summarized in Supplementary Table 2.

RRMS patients were diagnosed according to the 2017 revision of the McDonald criteria [87] (Supplementary Table 1). In cohort 1, 86.5% of patients experienced clinical symptoms meeting the criteria of an acute relapse and/or had active MRI lesions. In cohort 2, 25.0% of patients met the criteria of an acute relapse. None of the patients were treated with disease-modifying therapies prior to sampling. Supplementary Table 3 summarizes the basic demographic and clinical characteristics of RRMS patients.

Patients with primary CNS-DLBCL and IDH-wildtype gliomas (diffuse/anaplastic astrocytoma and glioblastoma) were diagnosed based on histopathological and molecular studies according to the current guidelines at the time of sampling [88–92]. In the basic mFC cohort, CNS-DLBCL and IDH-wildtype glioma patients, who have been diagnosed since 2012 were included. 18 patients with IDH-wildtype glioblastoma, who have been diagnosed since 2022 were included in the in-depth mFC cohort. None of the patients received tumor treatment prior to sampling. Basic demographic and clinical characteristics of all CNS-DLBCL and IDH-wildtype gliomas patients are shown in supplementary Table 4.

Diagnosis of SD was made according to the ICD-10 diagnostic criteria [93, 94]. SD patients had no comorbid neurological conditions and CSF analysis revealed an intrathecal white blood cell count (WBC) of < 5 cells/µl, intrathecal lactate levels < 2 mmol/l, an intact blood-CSF barrier (BCSFB) as indicated by the age-adjusted albumin ratio, no intrathecal immunoglobulin (Ig)-synthesis according to Reiber criteria, and an oligoclonal band pattern type 1, as described previously [95, 96].

The patient cohorts have partly been used before [95].

### Routine CSF analysis

Lumbar puncture and PB sampling were performed solely during clinical routine workup. CSF cells were counted in a Fuchs-Rosenthal chamber. Total protein, IgG, IgA, and IgM levels were measured by nephelometry. Protein and Ig levels were assessed in serum and a Reiber scheme was created to evaluate the integrity of the BCSFB and the quantity of intrathecal Ig synthesis. Isoelectric focusing and silver nitrate staining were performed to detect ocbs.

### MFC of PB and CSF samples

PB and CSF samples, obtained during clinical routine workup, were centrifuged for 15 min at 300 g. Samples were processed within one hour to ensure optimal sample quality. Supernatant was discarded, CSF cells were resuspended in parallel to 100 µl PB in 100 µl VersaLyse (Beckman Coulter, Brea, California, USA) and incubated for 10 min. Cells were washed and the fluorochrome-conjugated antibodies (Supplementary Table 5), diluted in flow cytometry buffer (FC buffer: Phosphate Buffered Saline (PBS)/Fetal Bovine Serum (FBS)/EDTA), were added. A washing step was performed, cells were centrifuged and re-suspended in FC buffer supplemented with 20 µl flow count fluorospheres (Beckman Coulter). Data acquisition was performed with a Navios flow cytometer (Beckman Coulter). Analysis was performed with ‘Kaluza Flow Cytometry Analysis’ version 2.1 (Beckman Coulter). Analyzed cell populations are summarized in supplementary Table 5. Percentages of cell populations were calculated and compared between groups.

### Isolation of PBMCs and mFC analysis

Blood samples were collected as part of the clinical routine workup. PBMCs were isolated from whole blood by Ficoll gradient with SepMate isolation tubes (StemCell Technologies) and were cryopreserved in liquid nitrogen.

In order to prepare samples for mFC, PBMCs were thawed and resuspended in FC buffer. Samples were centrifuged twice for 5 min at 1200 rpm and 4 °C and supernatant was discarded. FC buffer was added, and cells were transferred to a 96-well plate. Centrifugation was repeated and cells were resuspended in FC buffer containing a FcR Blocking Reagent (Miltenyi Biotec). Cells were incubated for 5 min at room temperature. Next, fluorochrome-conjugated antibodies (Supplementary Table 5), diluted in FC buffer, were added. Zombie Aqua Fixable Viability Kit (BioLegend) was used as a viability marker. Incubation was performed for 20 min at 4 °C. Afterwards, cells were washed, centrifuged and resuspended in FC buffer. For intracellular staining (FoxP3), the Foxp3/Transcription Factor Staining Buffer Set (eBioscience) was used following cell surface marker staining.

A CytoFLEX-S (Beckman Coulter) was used to acquire data. Manual gating was performed with the software ‘Kaluza Flow Cytometry Analysis’ version 2.1**.** The percentage of all living cells was calculated for every cell population and was compared between groups. Furthermore, unsupervised analysis was performed using the platform OMIQ from Dotmatics (www.omiq.ai, www.dotmatics.com). For this, compensated, pre-gated event data (panel 1: CD19^+^ B cells, panel 3: CD3^+^ lymphocytes, panel 5: monocytes (CD14^+^CD16^−^, CD14^+^CD16^+^, and CD14^−^CD16^+^), and panel 6: CD4^+^ T cells) were exported as csv files with the software ‘Kaluza Flow Cytometry Analysis’. The event data and the corresponding metadata were uploaded to the platform OMIQ. Opt-SNE plots including mFC data from glioblastoma and RRMS patients as well as from HC were created using the default parameters (max iterations = 1000, opt-SNE end = 5000, perplexity = 30, theta = 0.5, components = 2, random seed = 6230, verbosity = 25) (Supplementary Fig. 3). The algorithm PhenoGraph (K nearest neighbors = 20, distance metric = Euclidean, Louvain runs = 1, number of results = 1) was used for cluster identification. A clustered heatmap of concatenated files was created to visualize the median marker expression of each cluster (Supplementary Fig. 4 and 5). In total, 27 B cell (Bc) clusters, 63 T cell (Tc) clusters, and 24 monocyte clusters were identified (Supplementary Fig. 3). Bc cluster 2 and 10 as well as 5 and 9 were merged due to similarities in marker expression. Cluster Tc-II 22 was removed due to inconclusive marker expression.

### Data analysis, statistics, and visualization

‘R studio’ (2023.06.1) was used for data analysis and visualization. We performed principal component analysis (PCA) with the R package ‘FactorMineR’ (v2.11). Data were scaled in advance. Clustered heatmaps were created with the R package ‘pheatmap’ (v1.0.12). The group medians were calculated for every parameter in advance and row-wise scaling was performed. Violin plots with overlaying boxplots and volcano plots were created with the R package ‘ggplot2’ (v3.4.4). P-values were calculated using ANOVA with post-hoc Tukey HSD, if normality could be assumed based on Shapiro–Wilk test, otherwise Kruskal Wallis test with Dunn post hoc test (p-adjustment method: Benjamini–Hochberg) was used. A p-value of < 0.05 was considered statistically significant. For volcano plots, log2 fold change was computed for every parameter. P-values were plotted against the corresponding log2 fold change. All significant parameters are colored and labeled. Multiple linear regression was performed to adjust for differences in age and sex between groups. Parameters which did not remain significant after correcting for age and sex are shown in grey.

Sparse Partial Least Squares Discriminant Analysis (sPLS-DA) was applied to assess the performance of the PB mFC parameters, the CSF routine parameters, and the combination of CSF routine with PB and CSF mFC parameters to differentiate between groups. SPLS-DA was performed using the R package ‘mixOmics’ (v6.26.0). The ‘auroc’ function was used to calculate the Area Under the Curve (AUC) for the classification results obtained from sPLS-DA, higher values indicating better performance. Moreover, the contribution of the top ten variables on latent component 1 was visualized. The graphical abstract was created in BioRender (Räuber, S. (2024) https://BioRender.com/b72p208).

**Key resources table**

**Table:** 

Reagent or resource	Source	Identifier
Antibodies (Clone)		
CD3 (UCHT1)	Beckman Coulter	#A66327
CD3 (SK7)	BioLegend	#344804
CD3 (SK7)	BioLegend	#344807
CD4 (13B8.2)	Beckman Coulter	#IM2468
CD4 (SK3)	BioLegend	#344638
CD8 (B9.11)	Beckman Coulter	#A82791
CD8 (SK1)	BioLegend	#344723
CD8 (SK1)	BioLegend	#344730
CD14 (RM052)	Beckman Coulter	#B36297
CD14 (M5E2)	BioLegend	#301804
CD16 (3G8)	Beckman Coulter	#A66330
CD16 (3G8)	BioLegend	#302059
CD19 (J3-119)	Beckman Coulter	#B76283
CD19 (HIB19)	BioLegend	#302205
CD19 (HIB19)	BioLegend	#302226
CD45 (J33)	Beckman Coulter	#B36294
CD56 (N901)	Beckman Coulter	#A21692
CD56 (HCD56)	BioLegend	#318304
CD56 (HCD56)	BioLegend	#318327
CD138 (B-A38)	Beckman Coulter	#A40316
CD138 (DL-101)	BioLegend	#352306
HLA-DR (Immu-357)	Beckman Coulter	#B92438
HLA-DR (L243)	BioLegend	#307610
HLA-DR (L243)	BioLegend	#307629
IgD (IA6- 2)	BioLegend	#348222
CD5 (UCHT2)	BioLegend	#300629
CD11c (Bu15)	BioLegend	#337220
CD20 (2H7)	BioLegend	#302336
CD20 (2H7)	BioLegend	#302347
CD21 (Bu32)	BioLegend	#354922
CD24 (ML5)	BioLegend	#311122
CD25 (BC96)	BioLegend	#302629
CD27 (M-T271)	BioLegend	#356412
CD28 (CD28.2	BioLegend	#302908
CD38 (HB-7)	BioLegend	#356642
CD39 (A1)	BioLegend	#328218
CD40 (5C3)	BioLegend	#334337
CD40 (5C3)	BioLegend	#334322
CD45RA (HI100)	BioLegend	#304134
CD57 (QA17A04)	BioLegend	#393325
CD57 (QA17A04)	BioLegend	#393303
CD80 (2D10)	BioLegend	#305229
CD86 (BU63)	BioLegend	#374206
CD95 (DX2)	BioLegend	#305642
CD117 (104D2)	BioLegend	#313205
CD123 (6H6)	BioLegend	#306019
CD127 (A019D5)	BioLegend	#351333
CD127 (A019D5)	BioLegend	#351336
CD183 (G025H7)	BioLegend	#353704
CD192 (K036C2)	BioLegend	#357213
CD194 (L291H4)	BioLegend	#359419
CD195 (J418F1)	BioLegend	#359118
CD196 (G034E3)	BioLegend	#353417
CD197 (G043H7)	BioLegend	#353214
CD206 (15-2)	BioLegend	#321131
CD294 (BM16)	BioLegend	#350117
CD335 (9E2)	BioLegend	#331907
KLRG1 (14C2A07)	BioLegend	#368607
PD-1 (EH12.2H7)	BioLegend	#329906
Tim-3 (F38-2E2)	BioLegend	#345012
CTLA-4 (BNI3)	BioLegend	#369614
TIGIT (A15153G)	BioLegend	#372711
CCR10 (1B5)	BioLegend	#564771
FoxP3 (PCH101)	eBioscience	#12-4776-42
CX3CR1 (2A9-1)	BioLegend	#341626
Biological samples		
Relapsing–remitting multiple sclerosis (RRMS) patients (n = 148)	Department of Neurology with Institute of Translational Neurology, University Hospital Münster, Münster, Germany	NA
Relapsing–remitting multiple sclerosis (RRMS) patients (n = 20)	Department of Neurology, Medical Faculty and University Hospital Düsseldorf, Heinrich Heine University Düsseldorf, Düsseldorf, Germany	NA
Autoimmune limbic encephalitis (ALE) patients (n = 81)	Department of Neurology with Institute of Translational Neurology, University Hospital Münster, Münster, Germany	NA
Primary diffuse large B cell lymphoma of the CNS (CNS-DLBCL) patients (n = 9)	Department of Neurology with Institute of Translational Neurology, University Hospital Münster, Münster, Germany	NA
IDH-wildtype glioma patients (n = 33)	Department of Neurology with Institute of Translational Neurology, University Hospital Münster, Münster, Germany	NA
Glioblastoma patients (n = 18)	Department of Neurology, Medical Faculty and University Hospital Düsseldorf, Heinrich Heine University Düsseldorf, Düsseldorf, Germany	NA
Somatic symptom disorder (SD) patients (n = 110)	Department of Neurology with Institute of Translational Neurology, University Hospital Münster, Münster, Germany	NA
Healthy controls (n = 19)	Department of Neurology, Medical Faculty and University Hospital Düsseldorf, Heinrich Heine University Düsseldorf, Düsseldorf, Germany	NA
Chemicals, peptides, and recombinant proteins		
Zombie Aqua™ Fixable Viability Kit	BioLegend	#423102
VersaLyse	Beckman Coulter	#IM3648
Flow count fluorospheres	Beckman Coulter	#7547053
DPBS, no calcium, no magnesium	Thermo Fisher Scientific	#14190094
Fetal Bovine Serum (FBS)	Fisher Scientific	#17593595
SepMate isolation tubes	StemCell Technologies,	#85450
FcR Blocking Reagent	Miltenyi Biotec	#130-059-901
EDTA 0,5 M Ultra Pure	Thermo Fisher Scientific	#15575020
Critical commercial assays		
Foxp3/Transcription Factor Staining Buffer Set	eBioscience	#00-5523-00
Deposited data		
MFC and clinical data	ABCD-J data catalog	https://data.abcd-j.de/dataset/d55bbe2f-7e97-5e30-a03b-27119dd0c68d/1.0?tab=content
Software and algorithms		
Kaluza Flow Cytometry Analysis (v2.1)	Beckman Coulter	https://www.beckman.com/flow-cytometry/software/kaluza
OMIQ	Dotmatics	www.omiq.ai
R studio’ (2023.06.1)	R Core Team	https://www.r-project.org
R ggplot2 (v3.4.4)	Hadley Wickham	https://www.rdocumentation.org/packages/ggplot2
FactorMineR (v2.11)	Francois Husson	https://www.rdocumentation.org/packages/FactoMineR/versions/2.9
R pheatmap (v1.0.12)	Raivo Kolde	https://www.rdocumentation.org/packages/pheatmap/versions/1.0.10/topics/pheatmap
R mixOmics (v6.26.0)	F R, B G, A S, K-A LC (2017). “mixOmics: An R package for 'omics feature selection and multiple data integration.” *PLoS computational biology*, **13**(11), e1005752	http://www.mixOmics.org
BioRender	Shiz Aoki	https://www.biorender.com/
Other		
Navios flow cytometer	Beckman Coulter	NA
CytoFLEX-S	Beckman Coulter	NA

## Supplementary Information

Additional file 1.

Additional file 2.

## Data Availability

Data underlying this study are registered with the ABCD-J data catalog at https://data.abcd-j.de/dataset/d55bbe2f-7e97-5e30-a03b-27119dd0c68d/1.0?tab = content. Further information, resources, anonymized clinical and flow cytometry data can be requested via the catalog item and will be fulfilled by Nico Melzer (Nico.Melzer@med.uni-duesseldorf.de).
