# Supplementary material for: Flow cytometry identifies changes in peripheral and intrathecal lymphocyte patterns in CNS autoimmune disorders and primary CNS malignancies
Source: J Neuroinflammation. 2024 Nov 4;21:286. doi: 10.1186/s12974-024-03269-3 (PMC11536547; doi:10.1186/s12974-024-03269-3)
Supplement: Supplementary file 1 — Additional file 1. [file 12974_2024_3269_MOESM1_ESM.pdf]

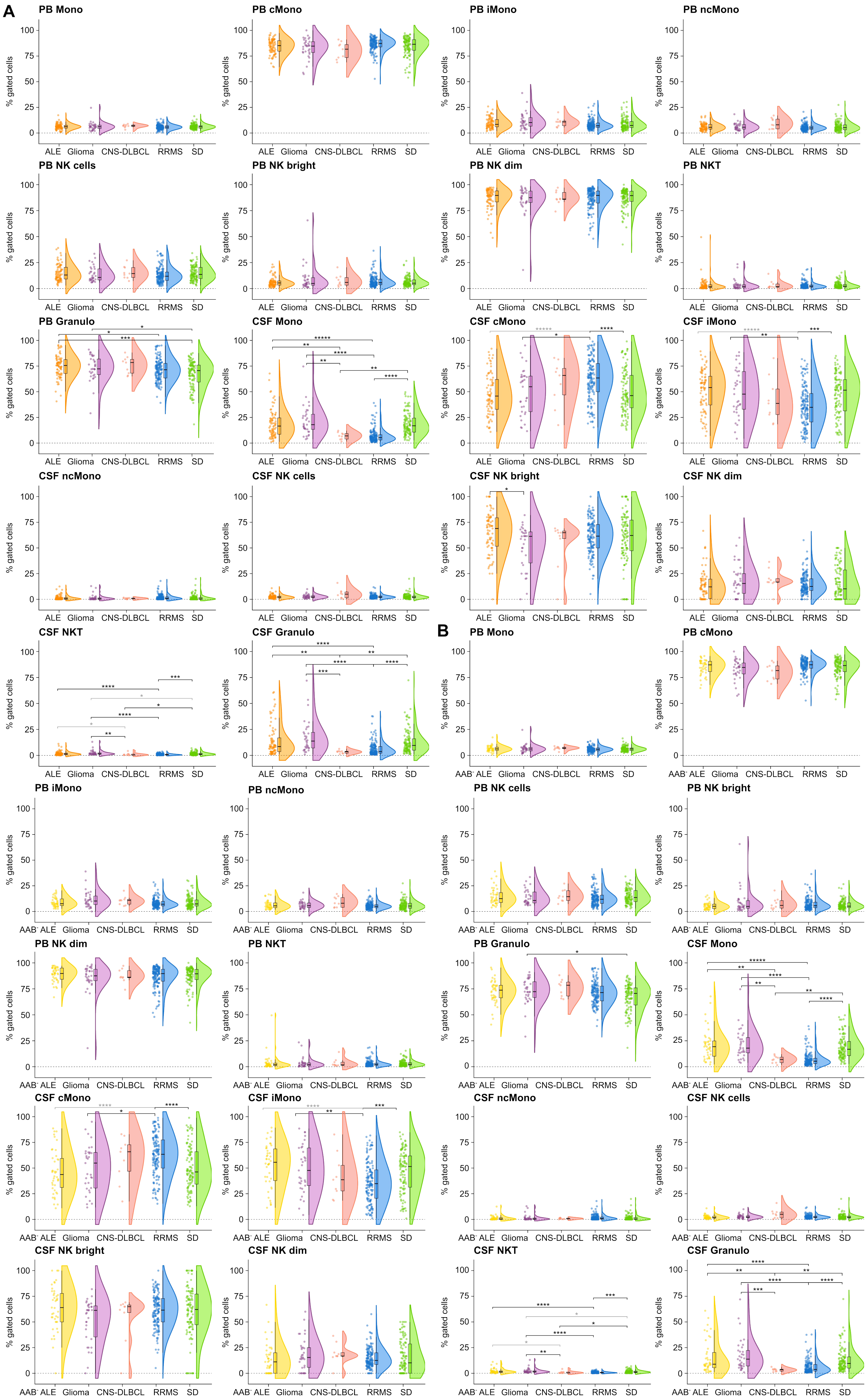

Supplementary figure 1 – Innate immune cell profiles of ALE, RRMS, IDH-wildtype glioma, CNS-DLBCL, and SD patients
Violin plots depicting the PB and CSF mFC parameters of ALE, RRMS, IDH-wildtype glioma, CNS-DLBCL, and SD patients. P-values were calculated by ANOVA with post-hoc Tukey HSD if normality could be assumed based on Shapiro–Wilk test, otherwise Kruskal Wallis test with Dunn post hoc test (p-adjustment method: Benjamini–Hochberg) was used. * p ≤ 0.05, ** p ≤ 0.01, *** p ≤ 0.001, **** p ≤ 0.0001.

AAB$^-$ ALE - antibody-negative ALE; ALE - autoimmune limbic encephalitis; cMono - classical monocytes; CNS - Central nervous system; CSF - cerebrospinal fluid; CNS-DLBCL - diffuse large B cell lymphoma of the central nervous system; IDH - isocitrate dehydrogenase; Granulo - granulocytes; iMono - intermediate monocytes; Lympho - lymphocytes; mFC - multidimensional flow cytometry; Mono - monocytes; ncMono; non-classical monocytes; NK - natural killer cells; NK bright - CD56^{bright}CD16$^{dim/-}$ natural killer cells; NK dim - CD56^{dim}CD16^{+} natural killer cells; NKT - Natural killer T cells; PB - peripheral blood; RRMS - relapsing remitting multiple sclerosis; SD - somatic symptom disorder; WT - wildtype.

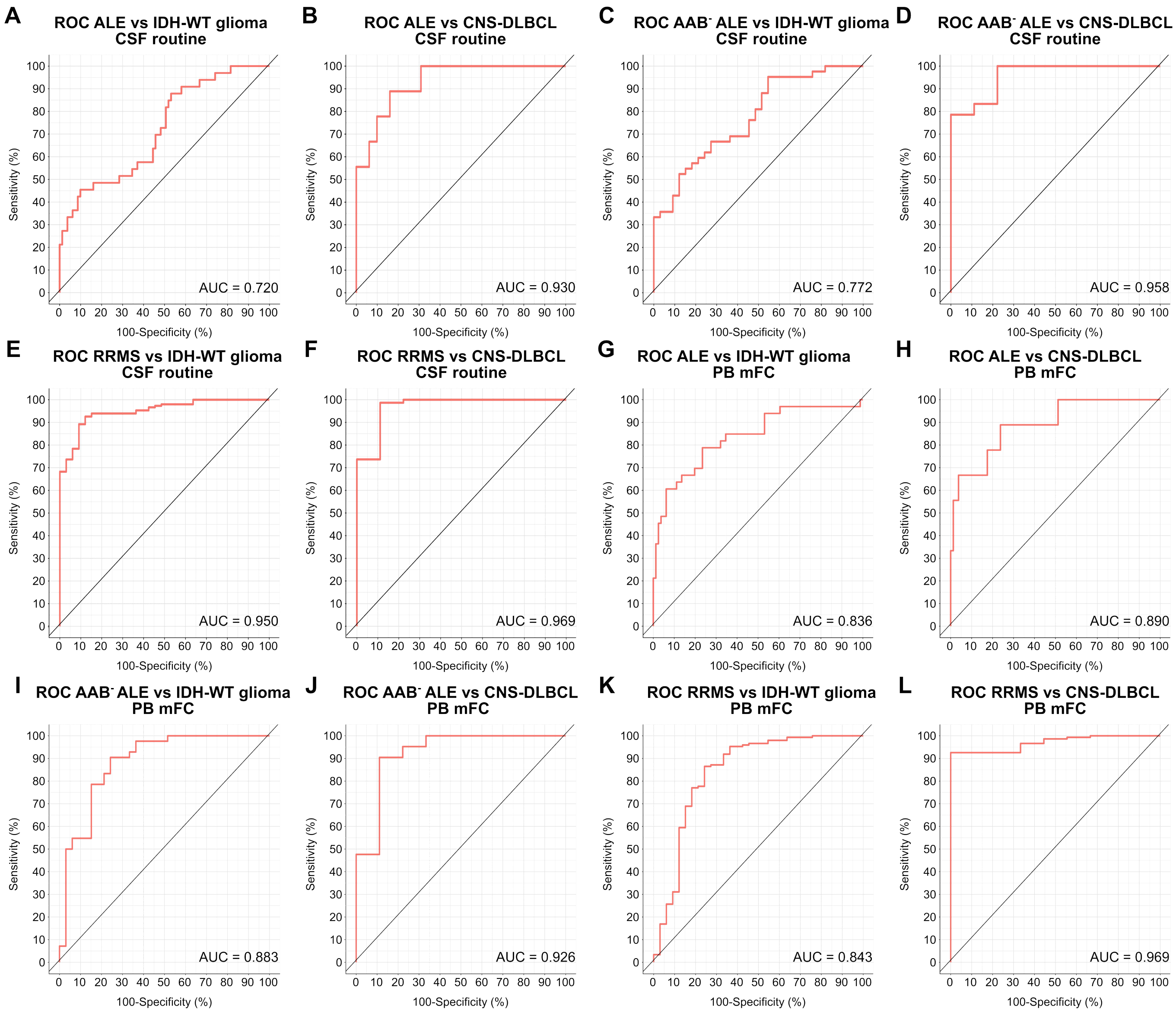

Supplementary figure 2 - CSF routine analysis or PB mFC alone are inferior to combined CSF routine, PB and CSF mFC analysis to distinguish ALE and RRMS from primary CNS tumors

A-F ROC analyses of the classification results obtained from sPLS-DA including CSF routine parameters. ALE patients (A and B), antibody-negative ALE patients (C and D), or RRMS patients (E and F) were compared to either IDH-WT glioma or CNS-DLBCL. **G-L** ROC analyses of the classification results obtained from sPLS-DA including PB mFC parameters. ALE patients (G and H), antibody-negative ALE patients (I and J), or RRMS patients (K and L) were compared to either IDH-WT glioma or CNS-DLBCL.

AAB^{-} ALE - antibody-negative ALE; ALE - autoimmune limbic encephalitis; AUC - Area under the curve; CNS - Central nervous system; CSF - cerebrospinal fluid; CNS-DLBCL - diffuse large B cell lymphoma of the central nervous system; IDH - isocitrate dehydrogenase; mFC - multidimensional flow cytometry; PB - peripheral blood; ROC - receiver operating characteristic; RRMS - relapsing remitting multiple sclerosis; sPLS-DA - Sparse Partial Least Squares Discriminant Analysis; WT - wildtype.

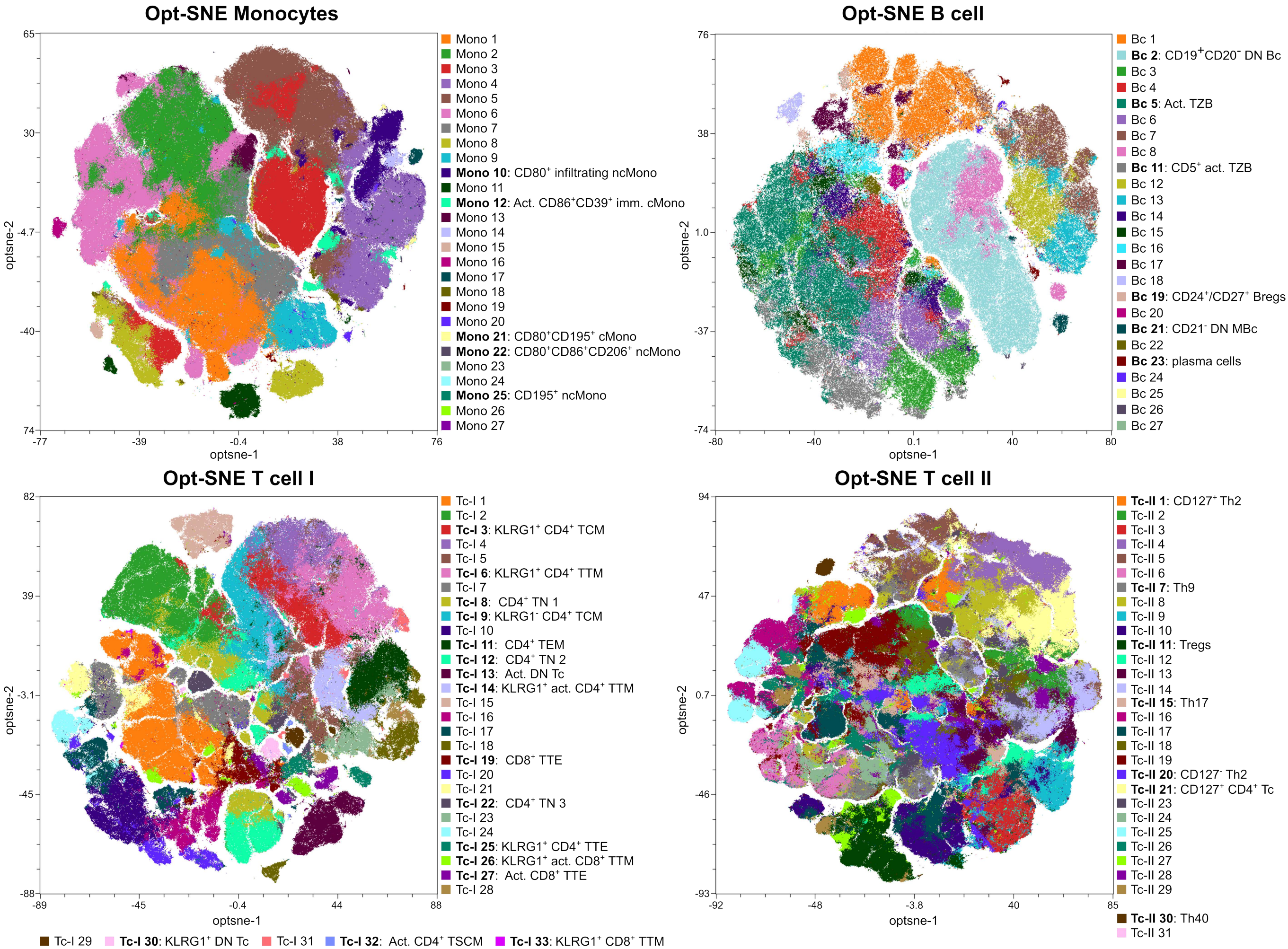

Supplementary figure 3 - Opt-SNE plots of cell clusters identified by PhenoGraph

Opt-SNE plots with B cell (mFC panel 1), T cell (mFC panels 3 & 6), and monocyte (mFC panel 5) clusters identified by PhenoGraph. Opt-SNE plots including mFC data from glioblastoma and RRMS patients as well as from HC were created with the platform OMIQ from Dotmatics (www.omiq.ai, www.dotmatics.com) using the default parameters (max iterations = 1000, opt-SNE end = 5000, perplexity = 30, theta = 0.5, components = 2, random seed = 6230, verbosity = 25). The algorithm PhenoGraph (K nearest neighbors = 20, distance metric = Euclidean, Louvain runs = 1, number of results = 1) was used for cluster identification.

Act. - activated; Bc - B cells; Bregs - B regulatory cells; cMono - classical monocytes; DN Bc - double negative ($CD27^-IgD^-$) B cells; DN Tc - double negative ($CD4^+CD8^+$) T cells; HC - healthy control; imm. - immature; iMono - intermediate monocytes; MBC - memory B cells; mFC - multidimensional flow cytometry; Mono - monocytes; ncMono; non-classical monocytes; RRMS - relapsing remitting multiple sclerosis; Tc - T cells; TCM - central memory T cells; TEM - effector memory T cells; ; Th - T helper cells; TN - naïve T cells; Tregs - regulatory T cells; TSCM - stem memory T cells; TTE - T terminal effector T cells; TTM - transitional memory T cells; TZB - transitional B cells.

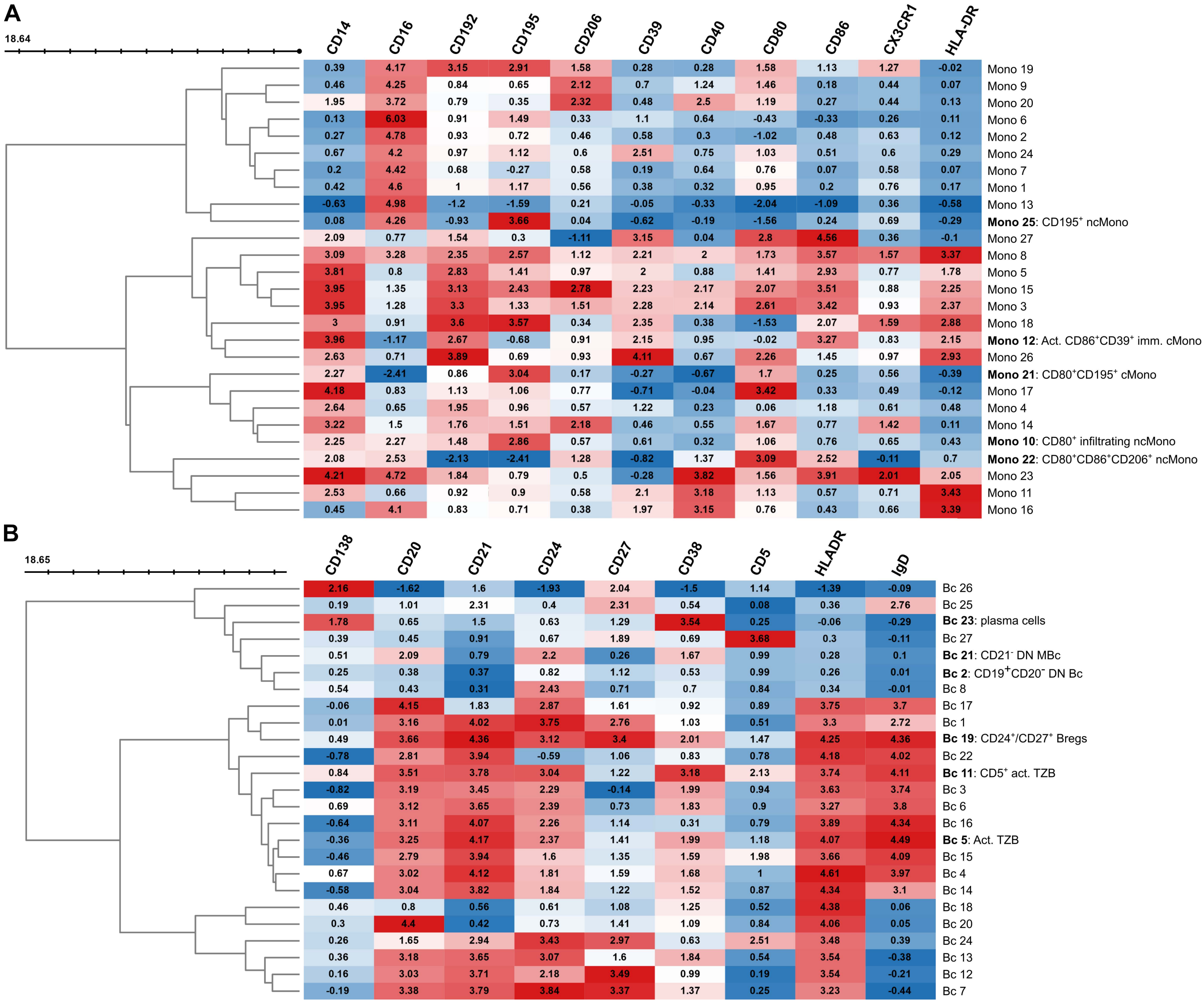

Supplementary figure 4 - Clustered heatmap of median marker expression of different cell clusters identified by PhenoGraph

A: Monocytes (mFC panel 5), **B:** B cells (mFC panel 1).

Act. - activated; Bc - B cells; Bregs - B regulatory cells; cMono - classical monocytes; DN Bc - double negative ($CD27^-IgD^-$) B cells; imm. - immature; iMono - intermediate monocytes; MBC - memory B cells; mFC - multidimensional flow cytometry; Mono - monocytes; ncMono; non-classical monocytes; TZB - transitional B cells.

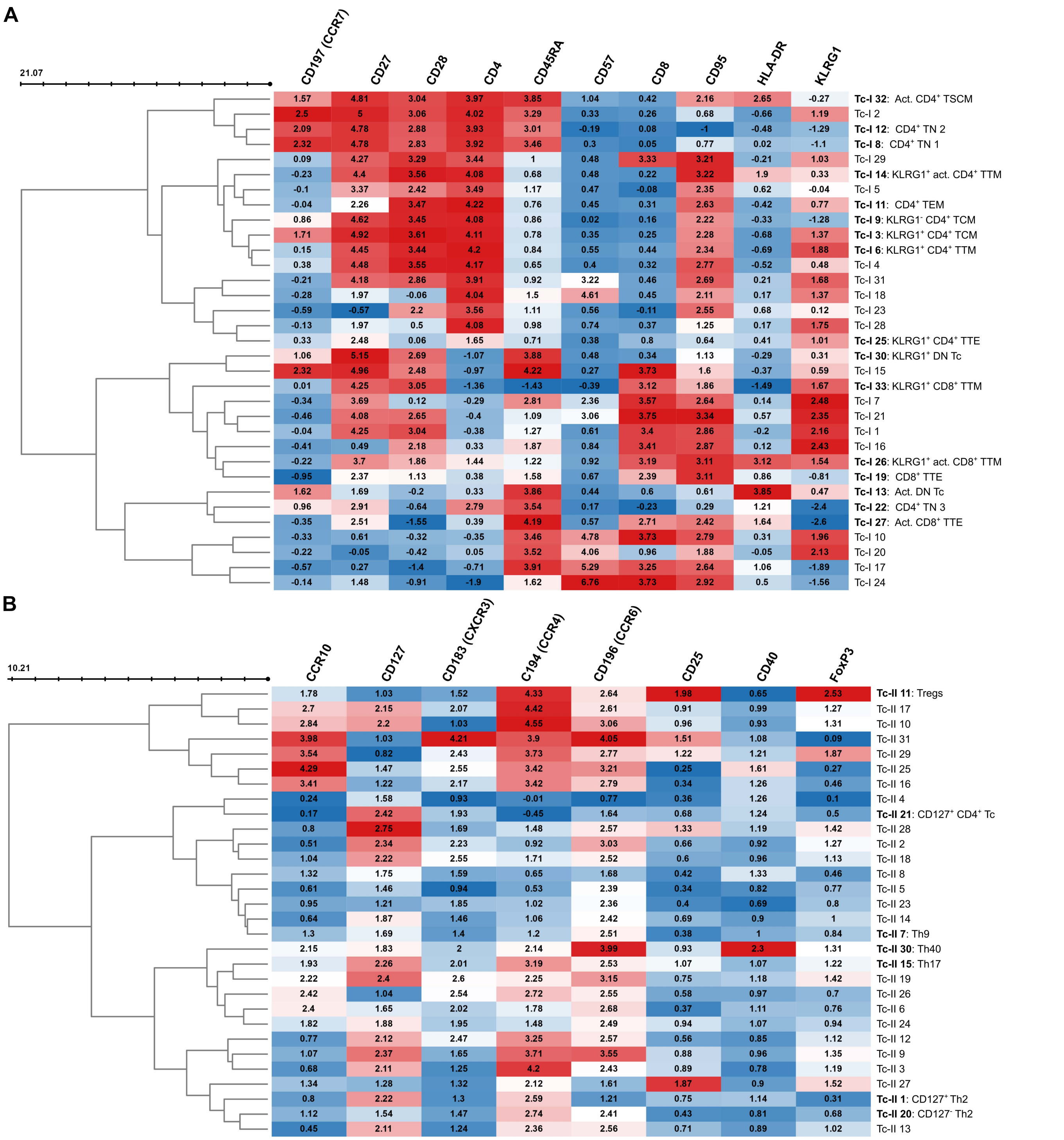

Supplementary figure 5 - Clustered heatmap of median marker expression of different cell clusters identified by PhenoGraph

A: T cell I (mFC panel 3), **B:** T cell II (mFC panel 6).

DN Tc - double negative (CD4$^+$CD8$^+$) T cells; mFC - multidimensional flow cytometry; Mono - monocytes; ncMono; non-classical monocytes; Tc - T cells; TCM - central memory T cells; TEM - effector memory T cells; Th - T helper cells; TN - naïve T cells; Tregs - regulatory T cells; TSCM - stem memory T cells; TTE - T terminal effector T cells; TTM - transitional memory T cells.

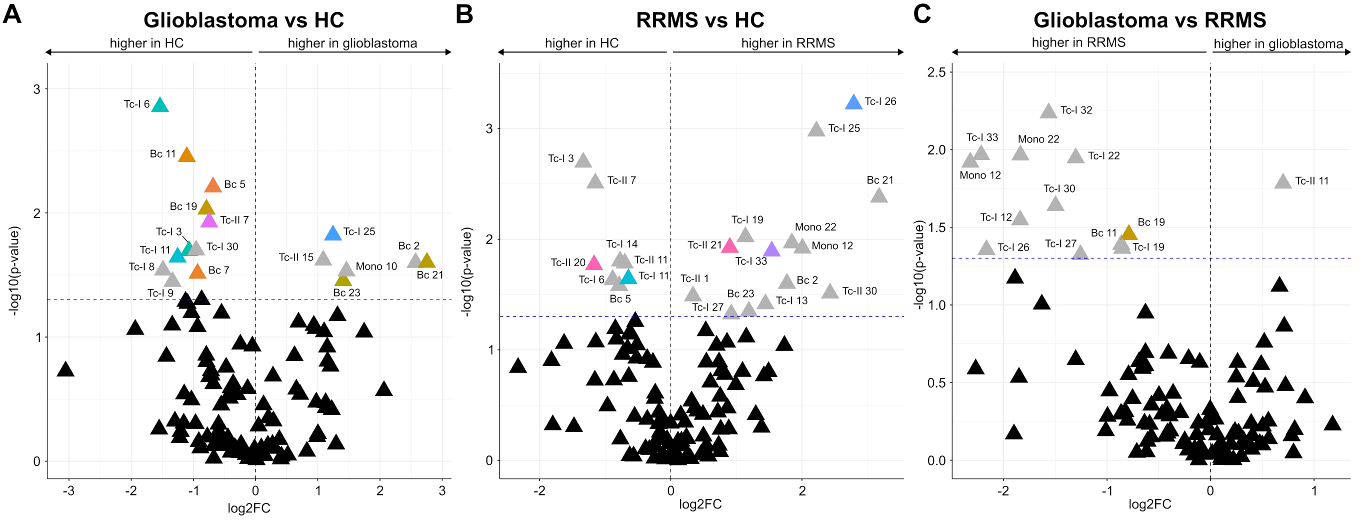

Bc 2: CD19$^+$CD20$^-$ DN Bc | **Bc 5**: Activated TZB | **Bc 11**: CD5$^+$ activated TZB | **Bc 19**: CD24$^+$/CD27$^+$ Bregs | **Bc 21**: CD21$^-$ DN memory Bc

Bc 23: plasma cells | **Tc-I 3**: KLRG1$^+$ CD4$^+$ TCM | **Tc-I 6**: KLRG1$^+$ CD4$^+$ TTM | **Tc-I 8**: CD4$^+$ TN 1 | **Tc-I 9**: KLRG1$^-$ CD4$^+$ TCM

Tc-I 11: CD4$^+$ TEM | **Tc-I 12**: CD4$^+$ TN 2 | **Tc-I 13**: Activated DN Tc | **Tc-I 14**: KLRG1$^+$ activated CD4$^+$ TTM | **Tc-I 19**: CD8$^+$ TTE

Tc-I 22: CD4$^+$ TN 3 | **Tc-I 25**: KLRG1$^+$ CD4$^+$ TTE | **Tc-I 26**: KLRG1$^+$ activated CD8$^+$ TTM | **Tc-I 27**: Activated CD8$^+$ TTE | **Tc-I 30**: KLRG1$^+$ DN Tc

Tc-I 32: Activated CD4$^+$ TSCM | **Tc-I 33**: KLRG1$^+$ CD8$^+$ TTM | **Mono 10**: CD80$^+$ infiltrating ncMono | **Mono 12**: Activated CD86$^+$CD39$^+$ immature cMono | **Mono 21**: CD80$^+$CD195$^+$ cMono

Mono 22: CD80$^+$CD86$^+$CD206$^+$ ncMono | **Mono 25**: CD195$^+$ ncMono | **Tc-II 1**: CD127$^+$ Th2 | **Tc-II 7**: Th9 | **Tc-II 11**: Tregs | **Tc-II 15**: Th17 | **Tc-II 20**: CD127$^-$ Th2 | **Tc-II 21**: CD127$^+$ CD4$^+$ Tc | **Tc-II 30**: Th40

Supplementary figure 6 – Volcano plots of cell clusters identified by unsupervised clustering

A-C Volcano plots illustrating the different cell clusters identified by PhenoGraph. The fold change of each single parameter between two groups is plotted against the corresponding p-value calculated by ANOVA with post-hoc Tukey HSD, if normality could be assumed based on Shapiro–Wilk test. Otherwise, Kruskal Wallis test with Dunn post hoc test (p-adjustment method: Benjamini-Hochberg) was used. Only significant parameters are labeled. Non-significant parameters are shown as black triangles. Parameters that did not remain significant after correction for age and sex are colored in grey. The following clusters are not shown because at least one median equals zero: A and B: Mono 21, Mono 25, Mono 26, Mono 27, and Tc-II 31; C: Mono 21, Mono 25, Mono 26, and Tc-II 31.

Bc - B cells; Bregs - B regulatory cells; cMono - classical monocytes; DN Bc - double negative (CD27$^-$IgD$^-$) B cells; DN Tc - double negative (CD4$^+$CD8$^+$) T cells; FC - fold change; HC - healthy control; mFC - multidimensional flow cytometry; Mono - monocytes; ncMono; non-classical monocytes; RRMS - relapsing remitting multiple sclerosis; Tc - T cells; TCM - central memory T cells; TEM - effector memory T cells; Th - T helper cells; TN - naïve T cells; Tregs - regulatory T cells; TSCM - stem memory T cells; TTE - T terminal effector T cells; TTM - transitional memory T cells; TZB - transitional B cells.
