# Supplementary material for: Flow cytometry identifies changes in peripheral and intrathecal lymphocyte patterns in CNS autoimmune disorders and primary CNS malignancies
Source: J Neuroinflammation. 2024 Nov 4;21:286. doi: 10.1186/s12974-024-03269-3 (PMC11536547; doi:10.1186/s12974-024-03269-3)
Supplement: Supplementary file 2 — Additional file 2. [file 12974_2024_3269_MOESM2_ESM.pdf]

Diagnostic criteria of ALE (Graus et al. [1])		
**Possible ALE**	**Probable ALE**	**Definitive ALE**
1. Subacute onset (< 3 month) of memory deficits, psychiatric symptoms or altered mental status	1. Subacute onset (< 3 month) of memory deficits, psychiatric symptoms or altered mental status	1. Subacute onset (< 3 months) of seizures, working memory deficits, or psychiatric symptoms
2. New focal CNS signs ± new onset seizures ± CSF pleocytosis ± hyperintense signal on T2-FLAIR MRI sequences highly restricted to one or both medial temporal lobes	2. CSF pleocytosis ± elevated CSF IgG index ± CSF-specific ocbs ± hyperintense signal on T2-FLAIR MRI sequences highly restricted to one or both medial temporal lobes ± biopsy showing inflammatory infiltrates and excluding other disorders	2. Hyperintense signal on T2-FLAIR MRI sequences highly restricted to both medial temporal lobes
3. Exclusion of reasonable differential diagnoses	3. Exclusion of reasonable differential diagnoses	3. CSF pleocytosis ± EEG with epileptiform or slow-wave activity involving the medial temporal lobes of both hemispheres
		4. Exclusion of reasonable differential diagnoses

Diagnostic criteria of RRMS (Thompson et al. [2])		
**Clinical attacks**	**Number of lesions with objective clinical evidence**	**Additional data needed for a diagnosis of multiple sclerosis**
≥ 2 clinical attacks	≥ 2	None
	1 (as well as clear-cut historical evidence of a previous attack involving a lesion in a distinct anatomical location)	None
	1	Dissemination in space demonstrated by an additional clinical attack implicating a different CNS site or by MRI
1 clinical attack	≥ 2	Dissemination in time demonstrated by an additional clinical attack or by MRI OR demonstration of CSF-specific ocbs
	1	Dissemination in space demonstrated by an additional clinical attack implicating a different CNS site or by MRI AND Dissemination in time demonstrated by an additional clinical attack or by MRI OR demonstration of CSF-specific ocbs

Supplementary table 1 - Diagnostic criteria of ALE and RRMS

1. Graus F, Titulaer MJ, Balu R, Benseler S, Bien CG, Cellucci T, et al. A clinical approach to diagnosis of autoimmune encephalitis. Lancet Neurol. 2016 Apr;15(4):391–404.
2. Thompson AJ, Banwell BL, Barkhof F, Carroll WM, Coetzee T, Comi G et al. Diagnosis of multiple sclerosis: 2017 revisions of the McDonald criteria. Lancet Neurol 2018; 17: 162–173.

ALE - autoimmune limbic encephalitis; CNS - central nervous system; CSF - cerebrospinal fluid; FLAIR - fluid-attenuated inversion recovery; Ig - immunoglobulin; MRI - magnetic resonance imaging; ocbs - oligoclonal bands; RRMS - relapsing-remitting multiple sclerosis

ID	Sex	Age	Ab	Ab target	Titer CSF	Titer serum	Grauss criteria	Signs and symptoms	Comorbidities	IMD ast	Plex/IA*	Cortisone*	ACD ast	SED ast	EEG abnormal
ALE1	f	27	-	-	-	-	definite	memory and attention deficits, seizures, emotional instability, depression	no	no	no	no	yes	no	yes
ALE2	f	64	DPPX	extra	1:1	1:1000	definite	memory deficits, autonomic dysfunction, diarrhea, weight loss, nystagmus, gait disturbance, vertigo, diplopia, depression	diabetes, hypothyroidism	no	no	no	no	no	no
ALE3	f	23	-	-	-	-	definite	memory deficits, seizures	s/p ALL	no	no	yes	yes	no	yes
ALE4	m	80	Hu	intra	1:100	1:1000	possible	memory deficits, disorientation, depression, speech disturbances, seizures	SCLC, afib, hypertension, PAD	no	no	no	no	no	yes
ALE5	m	47	GAD65	intra	1:100	1:1000	possible	memory deficits, seizures, emotional instability, anxiety	adrenal adenoma, hypertension, obesity, OSAS	no	no	no	yes	no	no
ALE6	m	65	CASPR2	extra	1:320	1:3200	possible	memory deficits, disorientation, seizures, formal thought disorder, infringing personal space	CTS, s/p stroke, diabetes, hypertension, COPD	no	no	no	yes	no	yes
ALE7	m	67	-	-	-	-	definite	memory deficits, seizures, depression	PNP, hypertension	no	no	no	no	no	yes
ALE8	f	17	-	-	-	-	possible	seizures, depression	hypothyroidism	no	no	no	yes	no	yes
ALE9	f	48	-	-	-	-	definite	memory deficits, disorientation, psychomotor retardation, seizures, psychosis, autonomic dysfunction	hypothyroidism, hypertension, bipolar disorder, obesity	no	no	yes	yes	no	yes
ALE10	m	66	-	-	-	-	probable	memory deficit, psychomotor retardation, depression	bipolar disorder, diabetes, hypertension, PNP	no	no	no	no	no	no
ALE11	f	53	GAD65	intra	1:10	1:320	definite	memory deficits, seizures	diabetes, rosacea	no	no	no	yes	no	no
ALE12	m	53	-	-	-	-	probable	memory deficits, depression	hypothyroidism	no	no	no	no	yes	yes
ALE13	m	50	LGI1	extra	neg.	1:32	possible	memory deficits, disorientation, seizures, anxiety, infringing personal space	asthma	no	no	no	yes	no	yes
ALE14	m	41	GAD65	intra	1:3.2	1:1000	possible	memory deficits, disorientation, seizures	no	no	no	no	yes	no	yes
ALE15	m	44	-	-	-	-	definite	memory deficits, seizures	no	no	no	no	yes	no	yes
ALE16	f	49	unknown	unknown	1:320	neg.	possible	memory deficits, seizures, depression	PNP, hashimoto thyroiditis, migraine, hypertension	no	no	no	yes	no	yes
ALE17	m	64	-	-	-	-	definite	memory deficits, disorientation, seizures	s/p PE	no	no	yes	yes	no	no
ALE18	f	59	Hu	intra	1:320	1:10000	definite	depression, anxiety, hallucinations	COPD, hypertension	no	no	no	no	yes	no
ALE19	m	52	-	-	-	-	definite	seizures, depression	asthma	no	no	no	yes	no	no
ALE20	m	67	-	-	-	-	definite	memory deficit, seizures, depression	s/p lymphoma, s/p testicular cancer	no	no	no	yes	no	yes
ALE21	m	70	-	-	-	-	possible	memory deficits, seizures, impulsive behavior, depression	s/p stroke, hypertension	no	no	no	no	no	yes
ALE22	m	53	-	-	-	-	definite	memory deficits, seizures, autonomic dysfunction	no	no	no	no	no	no	no
ALE23	f	43	GAD65	intra	1:10	1:100	definite	memory deficits, seizures, depression	no	no	no	no	no	no	yes
ALE24	m	51	GABA-A-R	intra	neg.	1:10	definite	memory deficits, seizures, psychosis, psychomotor retardation	s/p stroke, hypertension, adrenal adenoma, OSAS, asthma	no	yes	no	yes	no	yes
ALE25	f	61	-	-	-	-	possible	memory deficits, seizures	hypertension, hypothyroidism, afib, OSAS, buccal mucosa cancer	no	no	no	no	no	yes

ID	Sex	Age	Ab	Ab target	Titer CSF	Titer serum	Grauss criteria	Signs and symptoms	Comorbidities	IMD ast	Plex/IA*	Cortisone*	ACD ast	SED ast	EEG abnormal
ALE26	m	74	LGI1	extra	neg.	1:1000	definite	memory deficits, disorientation, seizures, speech disturbances, autonomic dysfunction, apraxia	CAD, s/p TIA, VaD, diabetes, hypertension	no	no	no	no	no	yes
ALE27	m	58	-	-	-	-	possible	seizures	depression, PNP	no	no	no	yes	no	yes
ALE28	f	60	GAD65	intra	1:100	1:100	possible	memory deficits, seizures, depression	s/p thymoma, hypertension, meningioma	no	no	no	yes	no	yes
ALE29	m	45	-	-	-	-	possible	memory deficits, seizures	hypertension	no	no	no	no	no	yes
ALE30	f	67	GAD65	intra	1:1	1:1000	definite	memory deficits, psychomotor retardation, emotional instability, seizures	s/p hashimoto thyroiditis, vitiligo	no	no	no	na	na	yes
ALE31	f	56	GABA-B-R	extra	neg.	1:10	definite	seizures	ITP	no	no	no	yes	no	yes
ALE32	f	22	-	-	-	-	definite	seizures	depression	no	no	no	yes	no	yes
ALE33	m	59	-	-	-	-	possible	memory deficits, seizures, psychosis, impulsive behavior, speech disturbances	OSAS, depression	no	no	no	yes	no	yes
ALE34	m	33	GAD65	intra	1:3.2	1:100	possible	psychomotor retardation, seizures, emotional instability, depression	chronic sinusitis	no	no	yes	yes	no	no
ALE35	m	66	CASPR2	extra	1:320	1:3200	possible	memory deficits, seizures	PAD, hypothyroidism	no	no	no	yes	no	yes
ALE36	m	53	-	-	-	-	definite	memory deficits, psychomotor retardation, depression, personality changes	hypertension, diabetes, obesity, COPD, OSAS, cardiomyopathy, hypercholesterolemia	no	no	no	no	no	no
ALE37	m	53	-	-	-	-	definite	memory deficits, seizures	no	no	no	no	na	na	yes
ALE38	f	49	-	-	-	-	definite	memory deficits	hypertension	no	no	no	yes	no	yes
ALE39	m	54	CASPR2	extra	1:320	1:3200	definite	memory deficits, seizures	no	no	no	no	no	no	yes
ALE40	f	55	-	-	-	-	possible	memory deficits, seizures, depression	PLMS	no	no	no	yes	no	yes
ALE41	m	44	-	-	-	-	definite	seizures, impulsive behavior, depression	thrombophilia, s/p PME and DVT, s/p testicular cancer, aortic stenosis	no	no	no	yes	no	yes
ALE42	f	59	GAD65	intra	neg.	1:100	definite	memory deficits, seizures, infringing personal space, depression	chronic headache, s/p PME and DVT, cholecystolithiasis, urolithiasis	no	no	no	yes	no	yes
ALE43	f	61	-	-	-	-	possible	memory deficits, emotional instability, depression	chronic tension-type headache	no	no	no	no	no	yes
ALE44	m	64	unknown	unknown	neg.	1:1000	definite	memory deficits, psychomotor retardation, seizures, emotional instability, depression, apraxia	CAD, emphysema, s/p duodenal ulcers	no	no	no	no	no	no
ALE45	f	71	LGI1	extra	neg.	1:100	definite	memory deficits, seizures, autonomic dysfunction	CAD, diabetes, hypertension, hypothyroidism	no	no	no	yes	no	yes
ALE46	m	49	LGI1	extra	1:1	1:1000	definite	memory deficits, seizures	no	no	no	yes	no	no	yes
ALE47	m	75	-	-	-	-	definite	memory deficits, psychomotor retardation, seizures, personality changes	PNP	no	no	no	no	no	yes
ALE48	f	76	LGI1	extra	1:1	1:1000	possible	memory deficits, seizures, depression, personality changes	hypertension, goiter	no	no	no	yes	no	yes

ID	Sex	Age	Ab	Ab target	Titer CSF	Titer serum	Grauss criteria	Signs and symptoms	Comorbidities	IMD ast	Plex/IA*	Cortisone*	ACD ast	SED ast	EEG abnormal
ALE49	m	56	GABA-B-R	extra	neg.	1:10	definite	memory deficits, psychomotor retardation, seizures, infringing personal space	no	no	no	no	yes	no	no
ALE50	m	58	-	-	-	-	definite	memory deficits, psychomotor retardation, seizures, agitation, anxiety, depression	PNP, CAD, PAD	no	no	no	yes	no	no
ALE51	m	22	-	-	-	-	definite	seizures	atopic dermatitis	no	no	no	no	no	yes
ALE52	m	66	LGI1	extra	1:1	1:100	possible	memory deficits, disorientation, impulsive behavior, psychosis, apraxia	Parkinson disease, Graves disease	no	no	yes	no	yes	yes
ALE53	f	71	-	-	-	-	possible	memory deficits, seizures, impulsive behavior, anxiety, autonomic dysfunction	hypertension, afib, goiter, renal angiomyolipoma	no	no	no	yes	no	yes
ALE54	m	78	LGI1	extra	1:1	1:100	definite	memory deficits, disorientation, psychomotor retardation, emotional instability, seizures, speech disturbances	hypertension, OSAS, heart failure	no	no	yes	yes	yes	no
ALE55	f	68	-	-	-	-	possible	seizures, impulsive behavior, anxiety	s/p DVT	no	no	yes	yes	no	yes
ALE56	f	61	-	-	-	-	possible	seizures, emotional instability, depression, speech disturbances	hypothyroidism	no	no	no	yes	yes	yes
ALE57	f	35	GAD65	intra	na	na	definite	memory deficits, seizures, depression, personality changes, speech disturbances	hypothyroidism, diabetes	no	no	no	yes	no	yes
ALE58	m	63	MaTa	intra	1:10	1:10	definite	memory deficits, disorientation, personality changes, depression, apraxia	heart failure, s/p PME and DVT, COPD, diabetes, hypertension, fatty liver	no	no	yes	yes	yes	yes
ALE59	m	41	VGKC	extra	neg.	1:64	definite	memory deficits, seizures, infringing personal space	amblyopia, strabismus, cataract	no	no	no	yes	no	yes
ALE60	f	57	GAD65	intra	1:10	1:1000	definite	memory deficits, seizures	PNP, hypertension	no	no	no	yes	no	yes
ALE61	m	53	Neurexin-3α	extra	1:1	1:100	definite	memory deficits, disorientation, seizures, depression, psychosis, parkinsonism	s/p DVT, hypertension	no	no	no	no	yes	no
ALE62	m	61	-	-	-	-	definite	memory deficits, seizures, depression	no	no	no	no	no	no	yes
ALE63	m	71	Hu, Sox Zic4, Yo	intra	na	na	possible	memory deficits, disorientation, agitation, seizures, ataxia, hypoesthesia, dysesthesia	SCLC, COPD, urothelial carcinoma	no	no	yes	yes	no	yes
ALE64	f	74	MaTa	intra	na	na	possible	memory deficits, impulsive behavior, psychomotor retardation, speech disturbances	s/p breast cancer, goiter, osteoporosis, pituitary adenoma	no	no	no	yes	no	yes
ALE65	f	64	-	-	-	-	possible	memory deficits, disorientation, seizures, depression, speech disturbances	s/p stroke, PNP, CAD, hypertension	no	no	no	yes	no	yes
ALE66	f	63	LGI1	extra	na	1:100	definite	memory deficits, disorientation, seizures, infringing personal space, formal thought disorder	no	no	no	yes	yes	yes	no
ALE67	f	54	LGI1	extra	neg.	1:10	definite	memory deficits, seizures, depression, personality changes	ovarian cancer, hypertension	no	no	yes	no	no	no
ALE68	m	74	-	-	-	-	definite	memory deficits, depression, hypoesthesia/paranesthesia	s/p stroke, s/p herpes zoster, hypertension, s/p MI, OSAS, MUGS	no	no	no	no	no	no

ID	Sex	Age	Ab	Ab target	Titer CSF	Titer serum	Grauss criteria	Signs and symptoms	Comorbidities	IMD ast	Plex/IA*	Cortisone*	ACD ast	SED ast	EEG abnormal
ALE69	f	68	-	-	-	-	definite	memory deficits, psychomotor retardation, seizures	s/p encephalitis of unknown origin, depressive episode, PNP, CTS, hypertension, goiter, s/p herpes zoster	no	no	no	yes	no	yes
ALE70	m	69	LGI1	extra	1:1	1:320	definite	memory deficits, disorientation, seizures, depression, speech disturbances, autonomic dysfunction	CAD, hypertension	no	no	no	yes	no	no
ALE71	m	53	-	-	-	-	probable	psychomotor retardation, speech disturbances	hypertension, s/p acute hepatitis B, s/p testicular cancer, CKid	no	no	no	no	no	yes
ALE72	f	48	-	-	-	-	possible	memory deficits, psychomotor retardation, seizures, depression, impulsive behavior, skew deviation	s/p stroke	no	no	no	yes	no	yes
ALE73	f	50	-	-	-	-	definite	memory deficits, seizures, infringing personal space, formal thought disorder, speech disturbances	PNP, s/p DVT, asthma, migraine, chronic gastritis, colorectal adenomas	no	no	no	yes	no	yes
ALE74	f	56	LGI1	extra	1:100	1:320	possible	memory deficits, disorientation, seizures, impulsive behavior, confabulations	s/p DVT	no	no	no	yes	no	yes
ALE75	m	76	DPPX	extra	1:32	1:10000	definite	memory deficits, disorientation, infringing personal space, depression, parkinsonism, gait disturbance	hypertension, s/p RCC	no	no	no	no	yes	no
ALE76	f	21	Yo, Ma/Ta	intra	na	na	definite	memory deficits, psychomotor retardation, seizures, infringing personal space	no	no	no	no	yes	no	yes
ALE77	m	57	-	-	-	-	definite	memory deficits, seizures, impulsive behavior, emotional instability	no	no	no	no	yes	no	yes
ALE78	m	61	-	-	-	-	definite	seizures, speech disturbances	AVNRT, s/p TIA, afib, hypertension, hypothyroidism	no	no	no	no	no	yes
ALE79	f	29	GFAP	intra	1:32	1:100	possible	memory deficits, formal thought disorder	fibromyalgia	no	no	yes	yes	no	yes
ALE80	m	43	GAD65	intra	1:10	1:1000	definite	memory deficits, seizures, autonomic dysfunction	asthma, s/p Graves disease	no	no	yes	yes	no	no
ALE81	m	27	-	-	-	-	definite	memory deficits, seizures, depression	s/p thyroid cancer, s/p depressive episode, obesity	no	no	no	yes	no	yes

Supplementary table 2 - Basic demographic and disease characteristics of ALE patients

Ab - antibody; ACD - anticonvulsive drugs; afib - atrial fibrillation; ALE - autoimmune limbic encephalitis; AVNRT - AV nodal reentrant tachycardia; CAD - coronary artery disease; CASPR2 - contactin-associated protein-like 2; CKid - chronic kidney disease; cMRI - cranial magnetic resonance imaging; COPD - Chronic obstructive pulmonary disease; CTS - carpal tunnel syndrome; DPPX - dipeptidyl-peptidase–like protein 6; DVT - deep vein thrombosis; EEG - electroencephalography; extra - extracellular; GABA-A-R - γ-aminobutyric acid A receptor; GABA-B-R - γ-aminobutyric acid B receptor; GAD65 - 65 kDa isoform of the glutamic acid decarboxylase; GFAP - glial fibrillary acidic protein; IA - immunoadsorption; intra - Intracellular; IMD - immunomodulatory drug; ITP - isolated thrombocytopenia; LGI1 - leucine-rich, glioma inactivated 1; MI - myocardial infarction; MUGS - monoclonal gammopathy of undetermined significance; OSAS - Obstructive sleep apnea syndrome; PAD - peripheral artery disease; PLEX - plasmapheresis; PLMS - Periodic limb movements of sleep; PME - pulmonary embolism; PNP - polyneuropathy; RCC - renal cell carcinoma; SCLC - small cell lung cancer; SED - sedative drugs; s/p - status post; TIA - Transient ischemic attack; VaD - vascular dementia; VGKC - voltage-gated potassium channel; ZIC4 - Zinc finger protein 4.

** within 4 weeks before sample taking*

Patient ID	Sex	Age	Comorbidities	Previous DMT	Plex/IA within 4 w p.t.s.	Cortisone within 4 w p.t.s.
RRMS1	f	49	na	None	No	No
RRMS2	f	61	na	None	No	No
RRMS3	f	38	na	None	No	No
RRMS4	f	26	na	None	No	No
RRMS5	f	25	na	None	No	No
RRMS6	f	32	na	None	No	No
RRMS7	f	45	na	None	No	No
RRMS8	f	23	na	None	No	No
RRMS9	f	33	na	None	No	No
RRMS10	f	22	na	None	No	No
RRMS11	f	25	na	None	No	No
RRMS12	f	47	na	None	No	No
RRMS13	m	17	na	None	No	No
RRMS14	f	45	na	None	No	No
RRMS15	f	51	na	None	No	No
RRMS16	m	24	na	None	No	No
RRMS17	f	29	na	None	No	No
RRMS18	f	30	na	None	No	No
RRMS19	f	29	na	None	No	No
RRMS20	m	23	na	None	No	No
RRMS21	f	28	na	None	No	No
RRMS22	m	29	na	None	No	No
RRMS23	m	31	na	None	No	No
RRMS24	m	23	na	None	No	No
RRMS25	f	30	na	None	No	No
RRMS26	f	24	na	None	No	No
RRMS27	m	19	na	None	No	No
RRMS28	m	28	na	None	No	No
RRMS29	m	25	na	None	No	No
RRMS30	f	47	na	None	No	No
RRMS31	f	47	na	None	No	No
RRMS32	f	21	na	None	No	No
RRMS33	f	36	na	None	No	No
RRMS34	f	30	na	None	No	No
RRMS35	f	40	na	None	No	No
RRMS36	m	25	na	None	No	No
RRMS37	f	28	na	None	No	No
RRMS38	f	55	na	None	No	No
RRMS39	f	24	na	None	No	No

Patient ID	Sex	Age	Comorbidities	Previous DMT	Plex/IA within 4 w p.t.s.	Cortisone within 4 w p.t.s.
RRMS40	f	25	na	None	No	No
RRMS41	f	32	na	None	No	No
RRMS42	f	23	na	None	No	No
RRMS43	m	42	na	None	No	No
RRMS44	f	25	na	None	No	No
RRMS45	f	44	na	None	No	No
RRMS46	f	31	na	None	No	No
RRMS47	m	22	na	None	No	No
RRMS48	f	18	na	None	No	No
RRMS49	f	19	na	None	No	No
RRMS50	m	24	na	None	No	No
RRMS51	f	37	na	None	No	No
RRMS52	f	52	na	None	No	No
RRMS53	f	21	na	None	No	No
RRMS54	m	55	na	None	No	No
RRMS55	f	52	na	None	No	No
RRMS56	f	16	na	None	No	No
RRMS57	f	31	na	None	No	No
RRMS58	f	32	na	None	No	No
RRMS59	m	27	na	None	No	No
RRMS60	f	16	na	None	No	No
RRMS61	f	32	na	None	No	No
RRMS62	f	38	na	None	No	No
RRMS63	f	25	na	None	No	No
RRMS64	m	30	na	None	No	No
RRMS65	f	50	na	None	No	No
RRMS66	f	43	na	None	No	No
RRMS67	f	28	na	None	No	No
RRMS68	f	35	na	None	No	No
RRMS69	m	35	na	None	No	No
RRMS70	f	29	na	None	No	No
RRMS71	f	41	na	None	No	No
RRMS72	f	21	na	None	No	No
RRMS73	f	45	na	None	No	No
RRMS74	f	42	na	None	No	No
RRMS75	f	27	na	None	No	No
RRMS76	m	25	na	None	No	No
RRMS77	f	18	na	None	No	No
RRMS78	f	20	na	None	No	No

Patient ID	Sex	Age	Comorbidities	Previous DMT	Plex/IA within 4 w p.t.s.	Cortisone within 4 w p.t.s.
RRMS79	f	29	na	None	No	No
RRMS80	f	23	na	None	No	No
RRMS81	m	18	na	None	No	No
RRMS82	m	45	na	None	No	No
RRMS83	f	24	na	None	No	No
RRMS84	m	28	na	None	No	No
RRMS85	f	24	na	None	No	No
RRMS86	f	21	na	None	No	No
RRMS87	f	47	na	None	No	No
RRMS88	f	42	na	None	No	No
RRMS89	f	41	na	None	No	No
RRMS90	f	22	na	None	No	No
RRMS91	m	47	na	None	No	No
RRMS92	f	26	na	None	No	No
RRMS93	m	29	na	None	No	No
RRMS94	m	20	na	None	No	No
RRMS95	f	27	na	None	No	No
RRMS96	f	21	na	None	No	No
RRMS97	f	43	na	None	No	No
RRMS98	f	34	na	None	No	No
RRMS99	f	37	na	None	No	No
RRMS100	f	25	na	None	No	No
RRMS101	m	29	na	None	No	No
RRMS102	f	41	na	None	No	No
RRMS103	f	44	na	None	No	No
RRMS104	m	44	na	None	No	No
RRMS105	f	50	na	None	No	No
RRMS106	f	25	na	None	No	No
RRMS107	f	36	na	None	No	No
RRMS108	f	26	na	None	No	No
RRMS109	f	46	na	None	No	No
RRMS110	f	33	na	None	No	No
RRMS111	m	32	na	None	No	No
RRMS112	f	34	na	None	No	No
RRMS113	m	49	na	None	No	No
RRMS114	f	31	na	None	No	No
RRMS115	f	31	na	None	No	No
RRMS116	f	30	na	None	No	No
RRMS117	f	42	na	None	No	No

Patient ID	Sex	Age	Comorbidities	Previous DMT	Plex/IA within 4 w p.t.s.	Cortisone within 4 w p.t.s.
RRMS118	m	27	na	None	No	No
RRMS119	m	50	na	None	No	No
RRMS120	m	25	na	None	No	No
RRMS121	f	48	na	None	No	No
RRMS122	f	16	na	None	No	No
RRMS123	f	49	na	None	No	No
RRMS124	m	40	na	None	No	No
RRMS125	m	43	na	None	No	No
RRMS126	f	27	na	None	No	No
RRMS127	m	15	na	None	No	No
RRMS128	m	33	na	None	No	No
RRMS129	m	52	na	None	No	No
RRMS130	f	32	na	None	No	No
RRMS131	m	33	na	None	No	No
RRMS132	f	26	na	None	No	No
RRMS133	f	32	na	None	No	No
RRMS134	f	18	na	None	No	No
RRMS135	m	28	na	None	No	No
RRMS136	m	21	na	None	No	No
RRMS137	f	15	na	None	No	No
RRMS138	f	23	na	None	No	No
RRMS139	f	20	na	None	No	No
RRMS140	m	26	na	None	No	No
RRMS141	f	24	na	None	No	No
RRMS142	f	46	na	None	No	No
RRMS143	f	47	na	None	No	No
RRMS144	f	28	na	None	No	No
RRMS145	f	29	na	None	No	No
RRMS146	f	35	na	None	No	No
RRMS147	f	24	na	None	No	No
RRMS148	f	22	na	None	No	No
RRMS149	m	39	Depressive adjustment disorder	None	No	No
RRMS150	f	39	Depression, anxiety disorder	None	No	No
RRMS151	f	57	Disc herniation	None	No	No
RRMS152	f	38	S/p infectious mononucleosis in adolescence	None	No	No
RRMS153	f	39	None	None	No	No
RRMS154	f	46	Hashimoto's thyroiditis	None	No	No
RRMS155	f	45	Vitamin D deficiency	None	No	No
RRMS156	f	57	Hashimoto's thyroiditis, migraine, arterial hypertension	None	No	No

Patient ID	Sex	Age	Comorbidities	Previous DMT	Plex/IA within 4 w p.t.s.	Cortisone within 4 w p.t.s.
RRMS157	f	44	None	None	No	No
RRMS158	f	44	Hashimoto's thyroiditis, ectopic pregnancy 2014, S/p. stomach reduction 01/2022, obesity	None	No	No
RRMS159	m	53	Moderate outlet stenosis of the left subclavian artery, Vitamin D deficiency, adrenal adenoma, hypercholesterolemia	None	No	No
RRMS160	m	54	Cataract, COPD, axonal PNP, arterial hypertension, s/p bladder cancer 2018, anxiety disorder	None	No	No
RRMS161	m	46	Vitamin D deficiency	None	No	No
RRMS162	m	56	S/P WPW syndrome, microangiopathy	None	No	No
RRMS163	m	41	Os vesalianum right, symptomatic epilepsy	None	No	No
RRMS164	m	36	Alcohol use disorder, hip osteoarthritis	None	No	No
RRMS165	m	53	spinal canal stenosis C6/7 with myelonedema, radiculopathy lumbar spine	None	No	No
RRMS166	f	55	Asthma, arterial hypertension, s/p disc herniation, s/p mammary surgery, PNP	None	No	No
RRMS167	f	54	cervical disc protrusions, scoliosis	None	No	No
RRMS168	f	57	S/p total knee replacement, s/p spinal surgery, s/p cholecystectomy, eutyreotic goiter, s/p resection of giant cell tumor of the right digitus III	None	No	No

Supplementary table 3 - Basic demographic and disease characteristics of RRMS patients

COPD - chronic obstructive pulmonary disease; DMT - disease modifying therapy; f - female; IA - immunoabsorption; m - male; PLEX - plasmapheresis; PNP - polyneuropathy; p.t.s. - prior to sampling; RRMS - relapsing-remitting multiple sclerosis; S/p - status post; w - weeks, WPW - Wolff–Parkinson–White

Patient-ID	Diagnosis	Sex	Age	IDH status	MGMT status	ATRX expr.	Previous tumor treatment	Steroids p.t.s	Comorbidities
Glio-1	Diffuse astrocytoma	f	25	WT	Methylated	No	No	No	Polymorphic psychiatric disorder with schizoid symptoms
Glio-2	Diffuse astrocytoma	f	56	WT	NA	Yes	No	No	S/p Alcohol use disorder
Glio-3	Glioblastoma	f	21	WT	Non-methylated	Yes	No	No	Symptomatic epilepsy
Glio-4	Diffuse astrocytoma	f	42	WT	NA	NA	No	No	None
Glio-5	Anaplastic astrocytoma	f	27	WT	Methylated	Yes	No	No	Hypothyroidism; s/p disc herniation, chronic gastritis
Glio-6	Glioblastoma	m	58	WT	Non-methylated	Yes	No	No	None
Glio-7	Anaplastic astrocytoma	m	71	WT	Methylated	Yes	No	No	None
Glio-8	Glioblastoma	f	67	WT	Methylated	NA	No	No	S/p acute renal failure, recurrent hyperkalemia and hyponatremia, hypothyroidism, normochromic, normocytic anemia, pAD stage IV, s/p femoral neck fracture, s/p left hip joint replacement
Glio-9	Anaplastic astrocytoma	m	68	WT	Methylated	NA	No	No	None
Glio-10	Glioblastoma	m	22	WT	Non-methylated	Yes	No	No	None
Glio-11	Anaplastic astrocytoma	m	68	WT	Methylated	Yes	No	No	Diabetes mellitus type II, s/p acute renal failure, coronary artery disease, s/p myocardial infarction, chronic venous insufficiency, s/p ulcus cruris with MRSA detection, OSAS, s/p depressive episode, s/p basal cell carcinoma
Glio-12	Glioblastoma	m	54	WT	Methylated	Yes	No	No	S/p testicular cancer
Glio-13	Glioblastoma	f	46	WT	Methylated	NA	No	No	None
Glio-14	Glioblastoma	f	54	WT	Methylated	NA	No	No	None
Glio-15	Anaplastic astrocytoma	m	80	WT	Non-methylated	Yes	No	No	Type C gastritis, arterial hypertension, s/p colon carcinoma
Glio-16	Anaplastic astrocytoma	m	45	WT	Non-methylated	No	No	No	S/p oligodendroglioma, structural epilepsy, pulmonary sarcoidosis, s/p meningitis in childhood
Glio-17	Glioblastoma	m	71	WT	Non-methylated	NA	No	No	S/p appendectomy, arterial hypertension, goiter
Glio-18	Glioblastoma	f	80	WT	NA	NA	No	No	Symptomatic epilepsy, arterial hypertension, compensated heart failure
Glio-19	Glioblastoma	f	57	WT	NA	NA	No	No	S/p. herpes zoster, s/p bilateral hearing loss, s/p papilloma resection

Patient-ID	Diagnosis	Sex	Age	IDH status	MGMT status	ATRX expr.	Previous tumor treatment	Steroids p.t.s	Comorbidities
Glio-20	Glioblastoma	m	63	WT	Non-methylated	Yes	No	No	S/p peripheral facial nerve palsy
Glio-21	Glioblastoma	m	45	WT	Non-methylated	NA	No	No	Symptomatic epilepsy, tumor of the distal esophagus without evidence of malignancy, s/p multiple lacunar cerebral infarcts, arterial hypertension, familial hypercholesterolemia, Diabetes mellitus type II
Glio-22	Glioblastoma	m	46	WT	Non-methylated	NA	No	No	None
Glio-23	Glioblastoma	m	63	WT	NA	NA	No	No	None
Glio-24	Glioblastoma	m	57	WT	Methylated	No	No	No	S/p symptomatic epileptic seizure, s/p resection of benign neoplasm of the lung, s/p DVT, s/p erysipelas
Glio-25	Glioblastoma	f	68	WT	Methylated	Yes	No	No	symptomatic epilepsy, irritable bowel syndrome, tendinitis
Glio-26	Anaplastic astrocytoma	f	20	WT	NA	Yes	No	No	None
Glio-27	Glioblastoma	m	76	WT	NA	Yes	No	No	Diabetes mellitus type 2, arterial hypertension, chronic pancreatitis, BPH, coronary artery disease
Glio-28	Glioblastoma	f	85	WT	Non-methylated	Yes	No	No	S/p pneumonia, symptomatic epilepsy, hypothyroidism, arterial hypertension, coronary artery disease, chronic renal insufficiency stage III, cerebral microangiopathy
Glio-29	Anaplastic astrocytoma	f	73	WT	Methylated	Yes	No	No	Parkinsonism, arterial hypertension, s/p bypass surgery
Glio-30	Glioblastoma	m	69	WT	Non-methylated	Yes	No	No	Pulmonary fibrosis/pulmonary emphysema, hypothyroidism, hashimoto's thyroiditis, COPD, s/p partial gastric resection, hypercholesterolemia
Glio-31	Anaplastic astrocytoma	m	60	WT	Non-methylated	Yes	No	No	S/p intestinal surgery after perforation
Glio-32	Glioblastoma	f	52	WT	Methylated	Yes	No	No	Structural epilepsy, s/p eradication of helicobacter pylori, thyroid nodules
Glio-33	Glioblastoma	f	43	WT	Non-methylated	Yes	No	No	Allergic rhinitis
Glio-34	Glioblastoma	m	82	WT	Non-methylated	Yes	No	No	S/p epileptic seizure, s/p spinal ischemia, s/p cholecystectomy due to cholecystitis, s/p laryngeal cancer, s/p resection of adrenocortical adenoma, steatosis hepatis, s/p prostate cancer, splenomegaly
Glio-35	Glioblastoma	m	47	WT	Non-methylated	Yes	No	Yes	None
Glio-36	Glioblastoma	m	69	WT	Methylated	Yes	No	No	Macrocytic hyperchromic polyglobulia, arterial hypertension, hypercholesterolemia, alcohol use disorder
Glio-37	Glioblastoma	m	79	WT	Methylated	Yes	No	No	Prostate cancer, s/p DVT, hypothyroidism, BPH, s/p, prostatitis

Patient-ID	Diagnosis	Sex	Age	IDH status	MGMT status	ATRX expr.	Previous tumor treatment	Steroids p.t.s	Comorbidities
Glio-38	Glioblastoma	f	60	WT	Methylated	Yes	No	No	Arterial hypertension, asthma, hypothyroidism
Glio-39	Glioblastoma	m	61	WT	Methylated	Yes	No	Yes	None
Glio-40	Glioblastoma	m	49	WT	Methylated	Yes	No	No	None
Glio-41	Glioblastoma	f	70	WT	Methylated	Yes	No	Yes	Arterial hypertension
Glio-42	Glioblastoma	m	59	WT	Non-methylated	Yes	No	No	Arterial hypertension
Glio-43	Glioblastoma	m	73	WT	Methylated	Yes	No	Yes	PAD stage IV on the left side, diabetes mellitus type II with diabetic foot syndrome, arterial hypertension, coronary artery disease, s/p aortocoronary venous bypass grafting, s/p myocardial infarction, carotid artery stenosis
Glio-44	Glioblastoma	m	55	WT	Methylated	Yes	No	Yes	Glaucoma, arterial hypertension, GERD
Glio-45	Glioblastoma	m	65	WT	Methylated	Yes	No	Yes	Coronary artery disease, pulmonary hypertension, hypertrophic cardiomyopathy, left bundle branch block, s/p PFO closure, s/p multiple cerebral infarcts, cerebral microangiopathy, carotid artery stenosis, COPD, OSAS, oesophagitis, type C gastritis, hyperuricemia, chronic renal insufficiency in stage II, s/p seizures
Glio-46	Glioblastoma	f	57	NA	NA	NA	No	No	Arterial hypertension, migraine, mitral valve insufficiency
Glio-47	Glioblastoma	m	67	WT	Non-methylated	Yes	No	No	Degenerative joint disease, metabolic syndrome, coronary artery disease, arterial hypertension, Barrett's esophagus, normocytic, normochromic anemia, s/p pneumonia, structural epilepsy
Glio-48	Glioblastoma	m	84	WT	Methylated	Yes	No	Yes	Arterial hypertension
Glio-49	Glioblastoma	m	86	WT	Methylated	Yes	No	Yes	Hyponatremia, arterial hypertension, normochromic, normocytic anemia, AV block I°, incomplete left bundle branch block, thiamine deficiency
Glio-50	Glioblastoma	m	76	WT	Non-methylated	Yes	No	Yes	Chronic pancreatitis, BPH, chronic renal insufficiency stage I, Schwartz-Bartter syndrome, s/p melanoma
Glio-51	Glioblastoma	m	72	WT	Methylated	Yes	No	No	Mitral valve insufficiency I°, carotid artery stenosis, suspected post-inflammatory solitary pulmonary lesion
CNS-DLBCL-1	CNS-DLBCL	m	69	NA	NA	NA	No	No	Symptomatic epilepsy, cerebral microangiopathy, anral gastritis, axial hiatal hernia, glycogen acanthosis, prostate cancer
CNS-DLBCL-2	CNS-DLBCL	m	63	NA	NA	NA	No	No	None
CNS-DLBCL-3	CNS-DLBCL	f	75	NA	NA	NA	No	No	Arterial hypertension, hyperthyroidism, constipation

Patient-ID	Diagnosis	Sex	Age	IDH status	MGMT status	ATRX expr.	Previous tumor treatment	Steroids p.t.s	Comorbidities
CNS-DLBCL-4	CNS-DLBCL	m	61	NA	NA	NA	No	No	None
CNS-DLBCL-5	CNS-DLBCL	f	70	NA	NA	NA	No	No	Osteoarthritis, s/p knee replacement, hallux valgus, bilateral hearing loss, crebral microangiopathy, dyslipoproteinemia, PNP, goiter
CNS-DLBCL-6	CNS-DLBCL	m	71	NA	NA	NA	No	No	Lesion of the adrenal gland, MUGS, arterial hypertension, gout, s/p cataract surgery
CNS-DLBCL-7	CNS-DLBCL	m	75	NA	NA	NA	No	No	Coronary artery disease with s/p stenting, hperlipoproteinemia, AV block I°, arterial hypertension
CNS-DLBCL-8	CNS-DLBCL	m	85	NA	NA	NA	No	No	Artial hypertension, Diabetes mellitus type 2, s/p central artery occlusion
CNS-DLBCL-9	CNS-DLBCL	m	90	NA	NA	NA	No	No	Atrial fibrillation, arterial hypertension, hiatal hernia, s/p surgery for spinal canal stenosis, prostate cancer, PNP, scoliosis

Supplementary table 4 - Basic demographic and disease characteristics of glioblastoma and CNS-DLBCL patients

ATRX - Alpha thalassemia/mental retardation syndrome X-linked; AV - atrioventricular block; BPH - Benign prostatic hyperplasia; CNS - central nervous system; CNS-DLBCL - diffuse large B cell lymphoma of the central nervous system; DM - Diabetes mellitus; DVT - deep vein thrombosis; expr. - expresion; GERD - Gastroesophageal reflux disease, Glio - glioblastoma; f - female; IDH - Isocitrate dehydrogenase; m - male; MGMT - Methylated-DNA-protein-cysteine methyltransferase; MUGS - Monoclonal gammopathy of unknown significance; OSAS - Obstructive sleep apnea syndrome; PAD - Peripheral arterial disease; PFO - Patent foramen ovale; p.t.s. - prior to sampling; S/p - status post

Panel	Tissue	Analyzed cohort	Antibodies (Clone, company)	Analyzed cell population
Basic	PB & CSF	Basic mFC cohort (81 ALE, 147 RRMS, 33 IDH-WT-Glio, 9 PCNSL)	CD3 (UCHT1), CD4 (13B8.2), CD8 (B9.11), CD14 (RM052), CD16 (3G8), CD19 (J3-119), CD45 (J33), CD56 (N901), CD138 (B-A38), and HLA-DR (Immu-357) (all Beckman Coulter)	B cells, Mono, cMono, iMono, ncMono, Lympho, total T cells, $CD4^{+}$ T cells, $CD4^{+}CD8^{+}$ T cells, $CD4^{+}HLA\text{-}DR^{+}$ T cells, $CD8^{+}$ T cells ($CD8^{+}$), $CD8^{+}HLADR^{+}$ T cells, plasma cells, $CD4^{+}/CD8^{+}$ ratio, Granulo, NK, NKT, NK dim, NK bright
In-depth 1 (B cell)	PBMCs	In-depth mFC cohort (20 RRMS, 18 GBM, 19 HC)	CD3 (SK7, Biolegend), CD56 (HCD56, Biolegend), CD14 (M5E2, Biolegend), HLA-DR (L243, Biolegend), CD19 (HIB19, Biolegend), IgD (IA6- 2, Biolegend), CD5 (UCHT2, Biolegend), CD38 (HB-7, Biolegend), CD138 (DL-101, Biolegend),CD20 (2H7, Biolegend), CD21 (Bu32, Biolegend), CD27 (M-T271, Biolegend), CD24 (ML5, Biolegend)	B cells, naive B cells, memory B cells, $CD24^{+}/CD27^{+}$ Breg, $CD24^{+}/CD38^{+}$ Breg, MZB, TZB, plasmablasts, plasma cells, MFIs: HLA-DR, CD5 on B cells
In-depth 2 (Exhaustion)	PBMCs	In-depth mFC cohort (20 RRMS, 18 GBM, 19 HC)	CD3 (SK7, Biolegend), CD8 (SK1, Biolegend), CD4 (SK3, Biolegend), CD19 (HIB19, Biolegend), CD20 (2H7, Biolegend), CD56 (HCD56, Biolegend), PD-1 (EH12.2H7, Biolegend), Tim-3 (F38-2E2, Biolegend), CTLA-4 (BNI3, Biolegend), TIGIT (A15153G, Biolegend)	NK, NKT, $CD4^{+}CD8^{+}$ Lympho, $CD20^{+}$ Lympho<br>MFIs: TIGIT, Tim-3, PD-1, CTLA-4 on $CD3^{+}$ Lympho, $CD4^{+}$ Lympho, $CD8^{+}$ Lympho, $CD4^{+}CD8^{+}$ Lympho, NK, NKT
In-depth 3 (T cell I)	PBMCs	In-depth mFC cohort (20 RRMS, 18 GBM, 19 HC)	CD3 (SK7, Biolegend), HLA-DR (L243, Biolegend), CD28 (CD28.2, Biolegend), CD27 (M-T271, Biolegend), KLRG1 (14C2A07, Biolegend), CD8 (SK1, Biolegend), CD4 (SK3, Biolegend), CD197 (G043H7, Biolegend), CD57 (QA17A04, Biolegend), CD45RA ('HI100, Biolegend), CD95 (DX2, Biolegend)	$CD3^{+}$ Lympho, $CD4^{+}$ Lympho, $CD8^{+}$ Lympho, $CD4^{+}$ naive T cells, $CD4^{+}$ TSCM, $CD4^{+}$ TCM, $CD4^{+}$ TEM, $CD4^{+}$ TTM, $CD4^{+}$ TTE, $CD8^{+}$ naive T cells, $CD8^{+}$ TSCM, $CD8^{+}$ TCM, $CD8^{+}$ TEM, $CD8^{+}$ TTM, $CD8^{+}$ TTE, $CD4^{+}CD8^{+}$ naive T cells, $CD4^{+}CD8^{+}$ TSCM, $CD4^{+}CD8^{+}$ TCM, $CD4^{+}CD8^{+}$ TEM, $CD4^{+}CD8^{+}$ TTM, $CD4^{+}CD8^{+}$ TTE, sen. Lympho, sen. $CD4^{+}$, senescent $CD8^{+}$<br>MFI: HLA-DR on $CD3^{+}$ Lympho, $CD4^{+}$ Lympho, $CD8^{+}$ Lympho, CD4+CD8+ Lympho
In-depth 4 (ILC & NK)	PBMCs	In-depth mFC cohort (20 RRMS, 18 GBM, 19 HC)	CD3 (SK7, Biolegend), CD14 (M5E2, Biolegend), CD19 (HIB19, Biolegend), CD335 (NKp46) (9E2, Biolegend), CD127 (A019D5, Biolegend), CD294 (CRTH2) (BM16, Biolegend), CD117 (104D2, Biolegend), CD11c (Bu15, Biolegend), CD16 (3G8, Biolegend), CD56 (HCD56, Biolegend), CD57 (QA17A04, Biolegend), CD123 (6H6, Biolegend)	ILC1, ILC2, ILC3, alternative ILC, NK cells, NK bright, NK dim, $CD57^{+}$ NK, DC, $CD11c^{+}CD56^{+}$

Panel	Tissue	Analyzed cohort	Antibodies (Clone, company)	Analyzed cell population
In-depth 5 (Mono)	PBMCs	In-depth mFC cohort (20 RRMS, 18 GBM, 19 HC)	CD14 (M5E2, Biolegend), CD39 (A1, Biolegend) CD80 (2D10, Biolegend), CD86 (BU63, Biolegend), CD40 (5C3, Biolegend), HLA-DR (L243, Biolegend), CD206 (15-2, Biolegend), CD16 (3G8, Biolegend), CD195 (J418F1, Biolegend), CD192 (K036C2, Biolegend), CX3CR1 (2A9-1, Biolegend)	cMono, iMono, ncMono, immature Mono, mature Mono, ag-Mono, non-ag-Mono, infil. Mono, CD80$^+$ cMono, CD80$^+$ iMono, CD80$^+$ ncMono, CD86$^+$ cMono, CD86$^+$ iMono, CD86$^+$ ncMono, CD206$^+$ cMono, CD206$^+$ iMono, CD206$^+$ ncMono, CD39$^+$ cMono, CD39$^+$ iMono, CD39$^+$ ncMono, CX3CR1$^+$ cMono, CX3CR1$^+$ iMono, CX3CR1$^+$ ncMono<br>MFI: HLA-DR, CD80, CD86, CD206, CD39, CXCR1, CD40 on cMono, iMono, ncMono
In-depth 6 (T cell II)	PBMCs	In-depth mFC cohort (20 RRMS, 18 GBM, 19 HC)	CD3 (SK7, Biolegend), CD8 (SK1, Biolegend), CD4 (SK3, Biolegend), CD183 (G025H7, Biolegend), CD25 (BC96, Biolegend), CCR10 (1B5, BD Biosciences), FoxP3 (PCH101, eBioscience), CD196 (G034E3, Biolegend) CD194 (L291H4, Biolegend), CD127 (A019D5, Biolegend), CD40 (5C3, Biolegend)	Th1, Th2, Th17, Th22, Th9, Th40, Treg

Supplementary table 5 - Overview of the used mFC panels

Ag - antigen-presenting; Altern - alternative; Breg - B regulatory cells; cMono - classical monocytes; CSF - cerebrospinal fluid; DC - dendritic cells; GBM - Glioblastoma multiforme; HC - healthy control; IDH-WT-Glio - IDH-wildtype glioma; ILC - innate lymphoid cells; iMono - intermediate monocytes; Infil. - infiltrating; Lympho - lymphocytes; mFC - multidimensional flow cytometry; MFI - mean fluorescence intensity; Mono - monocytes; MZB -Marginal zone like B cells; ncMono; non-classical monocytes; NK - natural killer cells; NK bright - CD56bright NK cells, NK dim - CD56dim NK cells, NKT - Natural killer T cells; PBMCs - peripheral blood mononuclear cells; PCNSL - primary CNS lymphoma ; RRMS - relapsing remitting multiple sclerosis; Sen - senescent; T - T cells; TCM - Central memory T cells; TEM - Effector memory T cells; Th - T helper cells; Treg - Regulatory T cells; TSCM - Stem memory T cells; TTE - terminal effector T cells; TZB - Transitional B cells.
